# Double body effect induced by integrating proprioceptive-vestibular and visual information

**DOI:** 10.1016/j.isci.2025.113819

**Published:** 2025-10-21

**Authors:** Caleb Liang, Wen-Hsiang Lin, Wei-Kai Liou, Bo-Yu Chen, Jie-Rong Lin, Yen-Tung Lee, Sufen Chen

**Affiliations:** 1 Department of Philosophy, National Taiwan University, Taipei, Taiwan; 2 Graduate Institute of Brain and Mind Sciences, National Taiwan University, Taipei, Taiwan; 3 Graduate Institute of Digital Learning and Education, National Taiwan University of Science and Technology, Taipei, Taiwan; 4 Department of Computer Science and Information Engineering, HungKuo Delin University of Technology, New Taipei City, Taiwan; 5 Institute of European and American Studies, Academia Sinica, Taipei, Taiwan; 6 Empower Vocational Education Research Center, National Taiwan University of Science and Technology, Taipei, Taiwan; 7 Optentia Research Focus Area, North-West University, Vanderbijlpark, South Africa

**Keywords:** Neuroscience, Behavioral neuroscience, Cognitive neuroscience, Human-computer interaction

## Abstract

Most studies in bodily self-consciousness were limited to the case of a single body. We performed VR experiments to test the hypothesis that it is possible for healthy subjects to experience the Double Body Effect—the experiential combination of double body ownership and double body-location. Under proprioceptive-vestibular and visual manipulations, participants wobbled involuntarily while watching two identical avatars doing exactly the same. The results showed that, in both the 1PP and 3PP conditions, it was indeed possible for healthy subjects to experience the Double Body Effect. This reveals that body ownership and body location are more flexible than most studies have considered so far. It also suggests that self-location and body-location are not the same experiences, and that the relation between self and body is more complicated than both traditional dualism and contemporary reductionism. Finally, our findings can serve as a preliminary model for understanding the perplexing phenomenology of heautoscopy.

## Highlights

• Healthy subjects can experience double body ownership and double body-location
• Double Body Effect can be induced by combining proprioceptive-vestibular and visual signals
• Double Body Effect can serve as a preliminary model for understanding heautoscopy

## Graphical abstract

**Figure:**
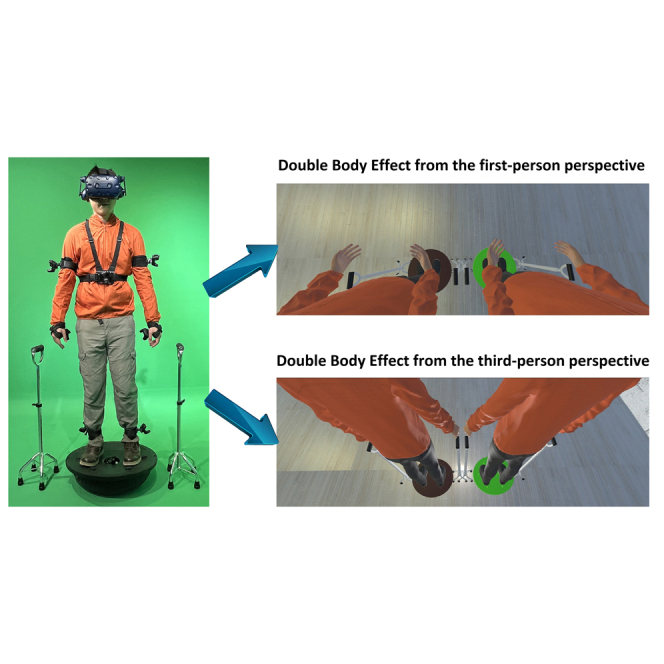

## Introduction

All human interactions with the world, including both the natural and social realms, are carried out via the body. One of the most basic bodily experiences is that each of us, from the first-person perspective (1PP), experiences a particular body at a specific location as our own. This involves two types of *bodily self-consciousness* (BSC): the sense of *body ownership* (the sense of a body-part or a full-body as *mine*) and the sense of *body-location* (the sense of where my body is located in space). These two types of BSC permeate all our perceptual experiences and sensorimotor activities, and they are closely connected to our sense of being a conscious self.^1^^,^^2^^,^^3^^,^^4^ Recent studies have suggested that the most basic sense of self that we have is the sense that each of us is a *subject of conscious experience*, and this fundamental form of self-consciousness is firmly rooted in bodily experiences.^5^^,^^6^ To be a subject is to have a 1PP as the origin and anchor of the ego-centric reference frame that structures one’s conscious experiences, and the 1PP is embedded in the body sustained by multisensory bodily signals.^7^^,^^8^^,^^9^ Thus, contrary to Cartesian dualism (the view that self and body are fundamentally distinct), most researchers across disciplines maintain that there is a tight (or even constitutive) link between self and body.^10^^,^^11^^,^^12^^,^^13^^,^^14^^,^^15^^,^^16^ However, exactly how tight is the link between self and body (especially the brain)? Are they more or less the and same thing, as contended by some contemporary reductionists?^17^^,^^18^ Or, is the relation between them actually more complicated than both traditional dualists and contemporary reductionists have suggested? A deeper understanding of BSC will be a crucial step in addressing these questions. In this study, we aim to provide a new approach to investigate BSC and hence the self-body relation by pursuing a key issue: Is it possible for us to have the experience of owning two bodies (*double body ownership*) located at two different places (*double body locations*)? Let us call this experiential combination the *Double Body Effect*.

The importance of this issue and how we intend to tackle it are due to four interdisciplinary considerations. First, studies on the rubber hand illusion (RHI) and full-body illusions have shown that, on the one hand, the sense of body ownership is constrained by certain anatomical, postural, and spatial-temporal factors^5^^,^^19^^,^^20^^,^^21^^,^^22^; on the other hand, it also exhibits a high degree of flexibility.^23^^,^^24^^,^^25^^,^^26^^,^^27^ However, given these constraining factors, exactly how flexible is the sense of body ownership? In addition, some neural explanations of body ownership have been suggested.^5^^,^^10^^,^^14^^,^^19^^,^^28^^,^^29^ If it is empirically possible for healthy subjects to experience double body ownership and double body-locations, what would this tell us about the relation between body and self? How may the Double Body Effect shed light on the current neural explanations of BSC? We propose to investigate the Double Body Effect because it enables us to address these important questions.

The possibility of experiencing dual ownership of body parts or whole bodies has been investigated by some previous studies. By viewing two fake hands and receiving tactile stimulations at the same time, it has been shown that subjects could feel as if they had two right hands.^24^^,^^25^^,^^30^ In Heydrich et al.,^27^ identical images of two bodies were generated such that participants watched two virtual bodies about 2 m in front of them. After receiving tactile stimulations on the back while watching the two virtual bodies being stroked in the corresponding areas, participants felt as if the two virtual bodies were their own. In Guterstam et al.,^26^ participants watched two identical bodies with the same postures from the 1PP. The sense of owning two bodies was induced under synchronous visual-tactile stimulations, supported by SCR evidence. When participants switched perspectives between 1PP and 3PP (third-person perspective), not only dual body ownership but also dual self-location were recorded in two different synchronous conditions. Based on these prior studies, we aim to make progress in the research of the Double Body Effect. Most of the previous studies were confined to either 1PP or 3PP set-ups, and all of them relied on visual-tactile stimulations. We therefore intended to investigate double body ownership and double body-location by integrating proprioceptive-vestibular and visual information from both 1PP and 3PP.

Second, one major approach in the research of BSC is to investigate bodily illusions. Most studies have used visual-tactile correlations to induce illusory experiences of body ownership and self-location.^21^^,^^26^^,^^31^^,^^32^^,^^33^^,^^34^ It was pointed out that when participants experienced illusory body ownership via visual-tactile manipulations, their internal bodily states were modulated and updated.^31^^,^^35^ Researchers also began to use other combinations of multisensory correlations in experiments, such as visual-motor,^36^^,^^37^ visual-proprioceptive,^38^ visual-vestibular,^39^ and visual-interoceptive^39^^,^^40^ manipulations. All of these suggest two things about BSC: first, proprioception (signals about limb position and movement), vestibular information (providing the sense of orientation and movement of our head and the direction of gravity), and interoception (somatosensory signals from viscera) are important for BSC. Second, these three types of internal bodily information, on the one hand, and exteroceptive perceptions, on the other, are not isolated from each other. Indeed, neurophysiological research has suggested that BSC depends on interactions between them.^7^^,^^14^^,^^29^^,^^41^^,^^42^^,^^43^^,^^44^ In this study, we focus on the central roles that proprioceptive and vestibular signals may play in bodily illusions, leaving interoception for another occasion.

Compared with the large body of literature that solely utilized visual-tactile stimulations, experiments that draw on proprioceptive-vestibular and visual correlations are rare. The previous works that considered proprioceptive and/or vestibular signals were mostly about variants of RHI.^38^^,^^45^^,^^46^^,^^47^ Only a few studies have experimented on full-body ownership using proprioceptive or vestibular manipulations, and all of them are limited to the case of a single body.^48^^,^^49^^,^^50^ We aim to advance this line of research by extending it to the case of double bodies. We applied VR technologies to conduct full-body experiments on the Double Body Effect that integrated exteroceptive perceptions (mainly vision) with proprioceptive and vestibular signals from within the body.^51^^,^^52^

Third, in addition to body ownership, *self-location* (the subjective feeling of *where I am* in space) is another basic form of BSC. In daily life, one’s sense of self-location overlaps with one’s sense of body-location. However, are they the same thing? Is the sense of where I am in space identical to the sense of where I feel my body is located? Addressing this issue involves examining the self-body relation from the angle of spatial awareness. Many researchers assume that self-location is identical to body-location, or at least do not distinguish between them.^16^^,^^53^^,^^54^^,^^55^^,^^56^^,^^57^ However, it is controversial whether this assumption is accurate. In the case of *out-of-body experience* (OBE), as Blanke and Mohr^58^ described, “During an OBE people seem to be awake and feel that their “self,” or center of awareness, is located outside of the physical body and somewhat elevated. It is from this elevated extrapersonal location that the subjects experience seeing their body and the world.” Here is an example cited by Blanke and Mohr^58^: “Suddenly it was as if he saw himself in the bed in front of him. He felt as if he were at the other end of the room, as if he were floating in space below the ceiling in the corner facing the bed from where he could observe his own body in the bed.” This suggests that it is possible for the sense of self-location to be dissociated from the sense of body-location. It also suggests that a different factor, that is, the experienced location and orientation of one’s first-person perspective (hereafter, *1PP-location*), may play an important role in the sense of self-location. Also, in the study of body-location by Huang et al.,^59^ participants watched their own body in front of them via a head-mounted display (HMD) connected to a stereo camera positioned behind them, and received tactile stimulations at the same time. By this manipulation, the participants’ sense of 1PP-location was transferred to the location of the camera. In one condition, the participants were instructed to walk straight ahead for about 2 m, while the camera remained stationary. In another condition, while the participants stood still, the camera was swiftly moved away from them for about 2 m. Results showed that body-location and 1PP-location were doubly dissociable, suggesting that they were different experiences. Given all these considerations, it is significant to investigate the relation between self-location and body-location as well as the relation between self-location and 1PP-location. Our experiments on the Double Body Effect provide useful ways to do exactly that.

Fourth, another approach to understanding BSC is to study pathological cases. For the purpose of this study, the most important and intriguing one is *heautoscopy*.^60^^,^^61^ This is a phenomenon that patients have the experience of seeing a *doppelgänger* of their own body in the extrapersonal space. Patients may feel uncertain about whether they are disembodied, and may be unsure about whether their self is dwelling within the physical body or within the autoscopic body. They may also have the experience of “bilocation” (the subjective feeling of being located at two different places simultaneously) and the experience of “perspective-switching” (the sense of their 1PP rapidly changing position between the physical and the autoscopic bodies).^1^^,^^16^ Although there are some case reports and studies regarding the clinical and neuroanatomical features about heautoscopy, currently we still know very little about this phenomenon.^57^^,^^58^^,^^62^^,^^63^ In fact, it is not even clear how to make sense of the puzzling phenomenology just mentioned. As we will see later, investigating whether healthy subjects can experience the Double Body Effect will contribute to a better understanding of heautoscopy.

In this study, we performed VR experiments to test the hypothesis that it is possible for healthy subjects to experience the Double Body Effect, that is, double body ownership *and* double body-location, by manipulations of proprioceptive-vestibular and visual signals. In our experimental design, while vision was still important, tactile stimulations were reduced to a minimum as compared to previous studies. Participants were immersed in a virtual gym where they saw and controlled a life-sized avatar either from the 1PP or from the 3PP. 1PP-experience refers to watching a virtual body (or bodies) from participants’ 1PP as if they directly look down at their own body. 3PP-experience refers to watching a virtual body (or bodies) from the 3PP as if participants look at someone else’s body from a distance. A motion tracker was attached to a Bosu ball (semicircular balance ball) such that it correlated perfectly with a virtual Bosu ball in the gym. Participants stepped onto the real Bosu ball while watching the avatar stepping onto the virtual one. This immediately caused the participants to wobble involuntarily to maintain balance. Except for Experiment 1, where only one avatar was present, participants saw the avatar splitting into two identical avatars, with their visual 1PP either located in the middle of the two avatars (Experiment 2), or located in the middle but about 60 cm behind the avatars (Experiment 3), or switching between the two avatars (Experiment 4). The constant wobbling triggered the participants’ proprioceptive sense and their vestibular system. The experimental results showed that the participants felt that both avatars were their own and that their body was simultaneously located at the places of the two avatars. We hence demonstrate that it is indeed possible for healthy subjects to experience the Double Body Effect, and that this effect can be induced by congruence of proprioceptive-vestibular and visual information.

## Results

Totally there were five experiments in this study (Table 1). We measured participants’ BSC by color-ball tests (CBT, measuring the sense of self-location; see Figure S1), skin conductance responses (SCR, physiological evidence for body ownership), and questionnaires (Tables 2 and 3) in Experiments 1–4. Experiment 5 involved only SCR measurements.

**Table 1:** Overview of experiments

Experiments	Variables			Measurements	Participants (n)
	Embodiment	Perspective	Synchronicity		
Exp. 1	Single body	1PP	Sync. v. Async.	Questionnaire, CBT & SCR	*n* = 40 (♂19)
Exp. 2	Double body	1PP	Sync. v. Async.	Questionnaire, CBT & SCR	*n* = 40 (♂21)
Exp. 3	Double body	3PP	Sync. v. Async.	Questionnaire, CBT & SCR	*n* = 40 (♂22)
Exp. 4	Double body	1PP Switch	Sync. v. Async.	Questionnaire, CBT & SCR	*n* = 40 (♂16)
Exp. 5	Double body	1PP	Sync. v. Mixed	SCR	*n* = 36 (♂10)

**Table 2:** Questionnaire of Experiment 1

Full-body ownership	Q1	It felt as if the virtual body was mine
	Q2	When I looked down, the body I saw seemed to be mine
Proprioceptive-vestibular referral	Q3	The wobbling that I felt seemed to be at the location of the virtual body
Agency	Q4	It felt as if I could control the virtual body
	Q5	It felt as if the movements of the virtual body were my movements
Body location	Q6	It felt as if my body was located at the place of the virtual body
Control question	Q7	It felt as if my body gradually became a refrigerator

**Table 3:** Questionnaire of Experiment 2–4

Double Body ownership	Q1	It felt as if both virtual bodies were mine
Double Proprioceptive-vestibular referral	Q2	The wobbling that I felt seemed to be at the locations of both virtual bodies
Double Agency	Q3	It felt as if the movements of both virtual bodies were my movements
Right Body ownership	Q4	It felt as if the right virtual body was also mine
Right Proprioceptive-vestibular referral	Q5	The wobbling that I felt seemed to be also at the location of the right virtual body
Right Agency	Q6	It felt as if the movements of the right virtual body were my movements
Left Body ownership	Q7	It felt as if the left virtual body was also mine
Left Proprioceptive-vestibular referral	Q8	The wobbling that I felt seemed to be also at the location of the left virtual body
Left Agency	Q9	It felt as if the movements of the left virtual body were my movements
Right Body location	Q10	It felt as if my body was also located at the place of the right virtual body
Left Body location	Q11	It felt as if my body was also located at the place of the left virtual body
Double Body location	Q12	It felt as if my body was simultaneously located at the places of the two virtual bodies
Control question	Q13	It felt as if my body gradually became a refrigerator

### Experiment 1: 1PP, single body conditions, Sync. vs. Async

**Figure 1:**
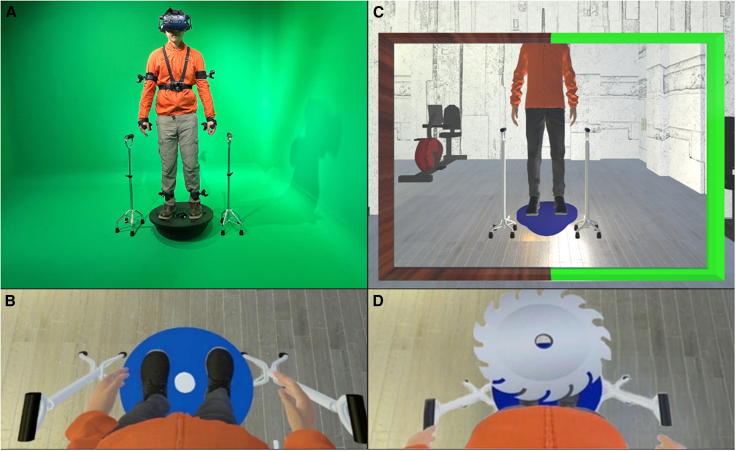
The set-up of Experiment 1 (A) The experimental set-up. The participants wore goggles and an orange jacket while standing on a Bosu ball. (B) When the participants looked down, they saw a life-sized avatar standing on a virtual Bosu ball. (C) When the participants looked straight ahead, they saw the avatar in a virtual mirror. (D) The participant’s skin conductance response (SCR) was measured after a virtual circular saw blade flew with noise toward the avatar.

**Figure 2:**
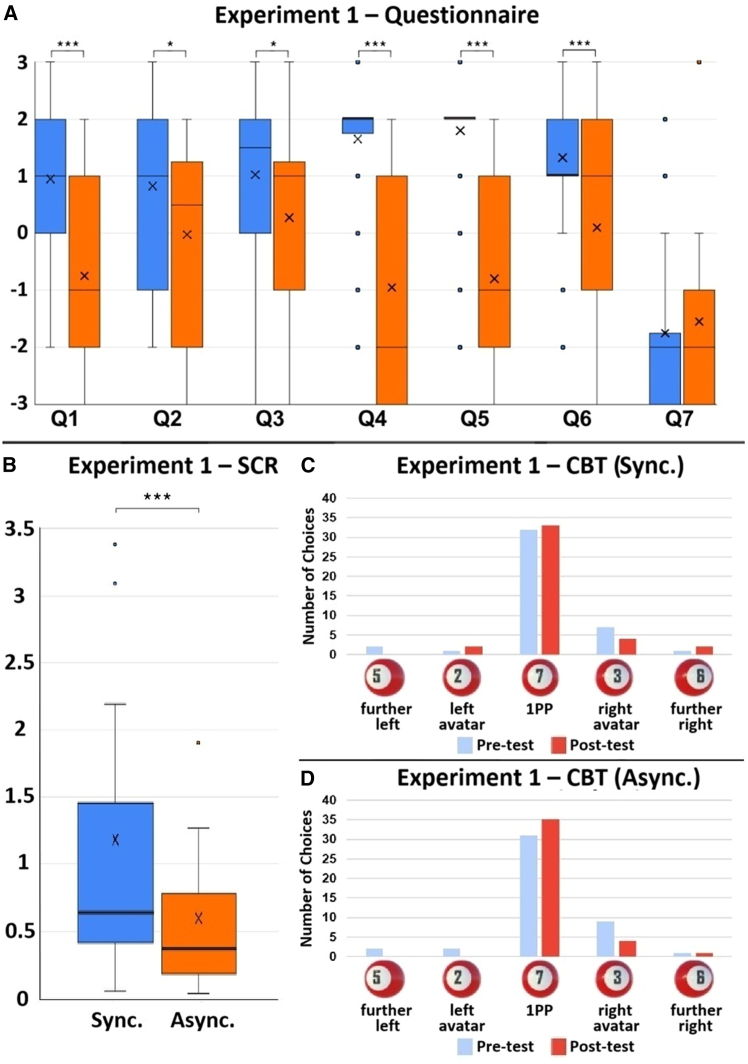
The results of Experiment 1. *N* = 40 (A) The Wilcoxon signed-rank test results of each questionnaire item. Q1: V = 541.5, *p* < 0.001, r = 0.702; Q2: V = 376.5, *p* = 0.011, r = 0.442; Q3: V = 349.5, *p* = 0.015, r = 0.425; Q4: V = 540, *p* < 0.001, r = 0.787; Q5: V = 580.5, *p* < 0.001, r = 0.810; Q6: V = 406.5, *p* < 0.001, r = 0.613; Q7: V = 101, *p* = 0.243, r = 0.140. (B) The SCR results. V = 678, *p* < 0.001, r = 0.570. The bold lines indicate the medians; upper and lower limits of the boxplot indicate the 75th and 25th percentiles. The error bars represent 1.5 IQR. Significance levels: ∗*p* < 0.05; ∗∗*p* < 0.01; ∗∗∗*p* < 0.001. (C) CBT results of the synchronous condition. Participants saw five 3D billiard balls with different numbers, appearing one at a time, spaced evenly apart in front of them. From left to right, the numbers were 5, 2, 7, 3, 6. Ball-7 was set directly in front of the participant’s visual 1PP. A pre-test was conducted before participants stepped onto the Bosu ball. A post-test was carried out after the experiments. (D) CBT results of the asynchronous condition.

In Experiment 1, when participants looked down, they saw from the 1PP a life-sized avatar standing on a virtual Bosu ball (Figure 1). In the synchronous condition, participants constantly and involuntarily wobbled on the real Bosu ball and watched the avatar simultaneously doing exactly the same on the virtual Bosu ball. In the asynchronous condition, participants did not step onto the Bosu ball, but stood on the ground the whole time. They saw the avatar from the 1PP but had no control of it at all. The box charts of each question and SCR values are shown in Figures 2A and 2B. The CBT showed that most participants felt as if they were at the location of the adopted visual 1PP (Figures 2C and 2D). The Wilcoxon signed-rank test showed significant differences between the synchronous and asynchronous manipulations on all questions except for the control question: Q1 (Body ownership) (V = 541.5, *p* < 0.001, r = 0.702), Q2 (Body ownership) (V = 376.5, *p* = 0.011, r = 0.442), Q3 (Proprioceptive-vestibular referral) (V = 349.5, *p* = 0.015, r = 0.425), Q4 (Agency) (V = 540, *p* < 0.001, r = 0.787), Q5 (Agency) (V = 580.5, *p* < 0.001, r = 0.810), Q6 (Body-location) (V = 406.5, *p* < 0.001, r = 0.613), Q7 (Control) (V = 101, *p* = 0.243, r = 0.140), and SCR (V = 678, *p* < 0.001, r = 0.570). These results suggest that in the synchronous condition, the participants felt as if the virtual body was their own, and as if their body was located at the place of the avatar.

### Experiment 2: 1PP, double body conditions, Sync. vs. Async

**Figure 3:**
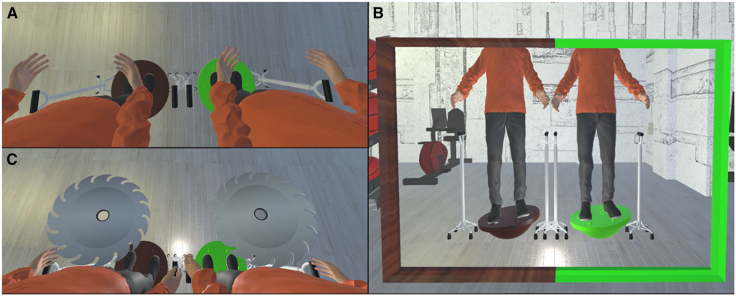
The set-up of Experiment 2 (A) When the participants looked down, they saw two life-sized avatars standing on virtual Bosu balls of different colors, and their visual 1PP was located in the middle of the two avatars. (B) When the participants looked straight ahead, they saw the avatars in a virtual mirror. (C) The participant’s SCR was measured after virtual circular saw blades flew toward both avatars.

**Figure 4:**
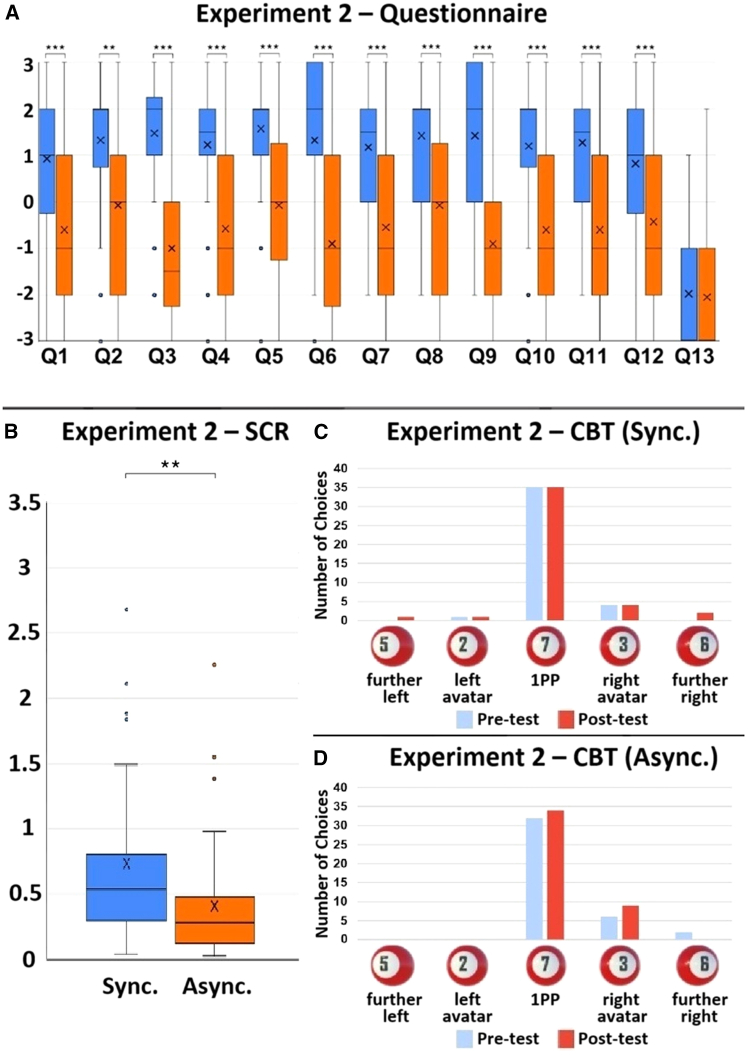
The results of Experiment 2. *N* = 40 (A) The Wilcoxon signed-rank test results of each questionnaire item. Q1: V = 513, *p* < 0.001, r = 0.603; Q2: V = 407.5, *p* = 0.002, r = 0.545; Q3: V = 579, *p* < 0.001, r = 0.807; Q4: V = 563, *p* < 0.001, r = 0.675; Q5: V = 421.5, *p* < 0.001, r = 0.621; Q6: V = 602, *p* < 0.001, r = 0.780; Q7: V = 541, *p* < 0.001, r = 0.689; Q8: V = 480.5, *p* < 0.001, r = 0.586; Q9: V = 610, *p* < 0.001, r = 0.786; Q10: V = 558, *p* < 0.001, r = 0.738; Q11: V = 435, *p* < 0.001, r = 0.693; Q12: V = 414.5, *p* = 0.001, r = 0.561; Q13: V = 50, *p* = 0.775, r = 0.046. (B) The SCR results. V = 640, *p* = 0.002, r = 0.489. The bold lines indicate the medians; upper and lower limits of the boxplot indicate the 75th and 25th percentiles. The error bars represent 1.5 IQR. Significance levels: ∗*p* < 0.05; ∗∗*p* < 0.01; ∗∗∗*p* < 0.001. (C) CBT results of the synchronous condition. Participants saw five 3D billiard balls with different numbers, appearing one at a time, spaced evenly apart in front of them. From left to right, the numbers were 5, 2, 7, 3, 6. Ball-5 was located to the front-left of the left avatar. Ball-2 was directly in front of the left avatar. Ball-7 was in front of the participant’s visual 1PP. Ball-3 was located directly in front of the right avatar. Ball-6 was to the front-right of the right avatar. A pre-test was conducted before participants stepped onto the Bosu ball. A post-test was carried out after the experiments. (D) CBT results of the asynchronous condition.

After participants stepped onto the Bosu ball, they saw the avatar splitting into two identical avatars, with their visual 1PP located in the middle of the two (Figure 3). In the synchronous condition, participants wobbled on the real Bosu ball and watched the two avatars simultaneously doing exactly the same on the virtual Bosu balls. In the asynchronous condition, participants did not step onto the virtual Bosu ball but stood on the ground the whole time. They saw the avatars from the 1PP but had no control over them. The box charts of each question and SCR values are shown in Figures 4A and 4B. The CBT showed that most participants felt as if they were located at the place of the adopted visual 1PP (Figures 4C and 4D). Significant differences were observed between the synchronous and asynchronous conditions on all questions except for the control question: Q1 (double Body ownership) (V = 513, *p* < 0.001, r = 0.603), Q2 (double Proprioceptive-vestibular referral) (V = 407.5, *p* = 0.002, r = 0.545), Q3 (double Agency) (V = 579, *p* < 0.001, r = 0.807), Q4 (right Body ownership) (V = 563, *p* < 0.001, r = 0.675), Q5 (right Proprioceptive-vestibular referral) (V = 421.5, *p* < 0.001, r = 0.621), Q6 (right Agency) (V = 602, *p* < 0.001, r = 0.780), Q7 (left Body ownership) (V = 541, *p* < 0.001, r = 0.689), Q8 (left Proprioceptive-vestibular referral) (V = 480.5, *p* < 0.001, r = 0.586), Q9 (left Agency) (V = 610, *p* < 0.001, r = 0.786), Q10 (right Body-location) (V = 558, *p* < 0.001, r = 0.738), Q11 (left Body-location) (V = 435, *p* < 0.001, r = 0.693), Q12 (double Body-location) (V = 414.5, *p* = 0.001, r = 0.561), Q13 (Control) (V = 50, *p* = 0.775, r = 0.046), and SCR (V = 640, *p* = 0.002, r = 0.489). These results suggest that in the synchronous condition, participants experienced from the 1PP both double body ownership and double body-locations with respect to the avatars.

### Experiment 3: 3PP, double body conditions, Sync. vs. Async

**Figure 5:**
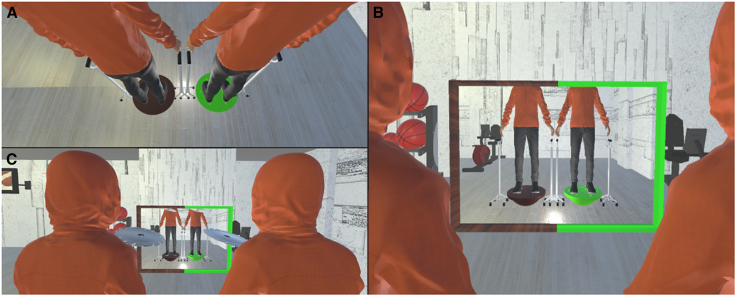
The set-up of Experiment 3 (A) When the participants looked down, they saw two life-sized avatars standing on virtual Bosu balls of different colors, and their visual 1PP was located in the middle and about 60 cm behind the two avatars. (B) When they looked straight ahead, they would see the back-sides of the two avatars as well as the front-sides of the two avatars in a virtual mirror. (C) The participant’s SCR was measured after virtual circular saw blades flew toward both avatars.

**Figure 6:**
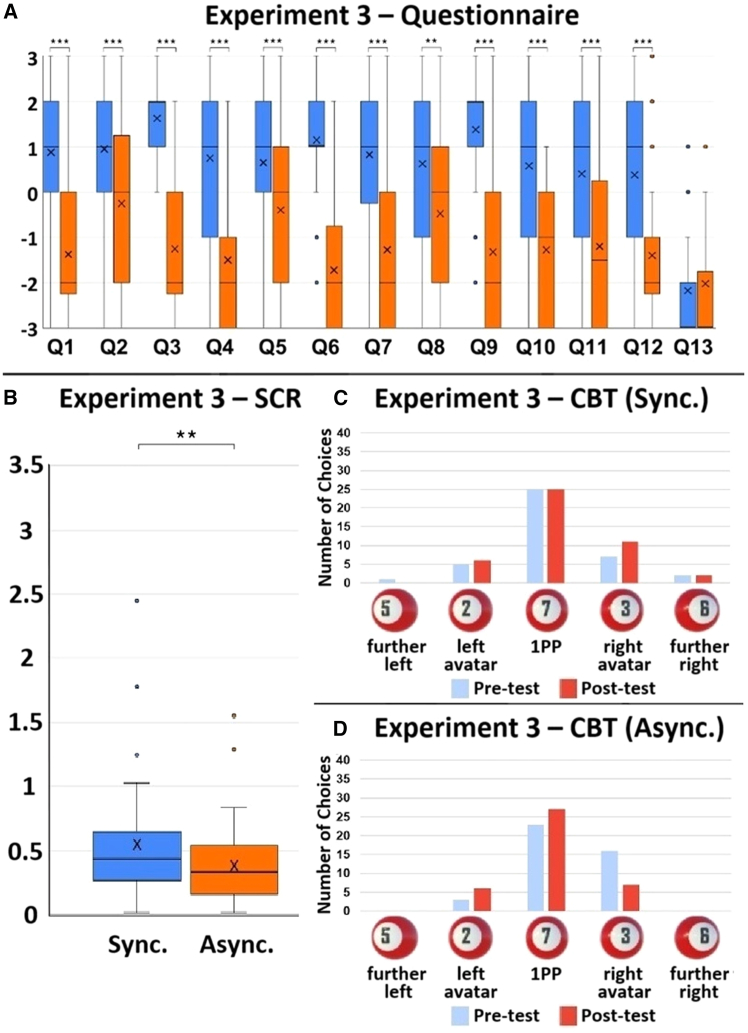
The results of Experiment 3. *N* = 40 (A) The Wilcoxon signed-rank test results of each questionnaire item. Q1: V = 648, *p* < 0.001, r = 0.728; Q2: V = 357, *p* < 0.001, r = 0.541; Q3: V = 662, *p* < 0.001, r = 0.841; Q4: V = 513, *p* < 0.001, r = 0.773; Q5: V = 414, *p* = 0.001, r = 0.521; Q6: V = 777, *p* < 0.001, r = 0.784; Q7: V = 598, *p* < 0.001, r = 0.761; Q8: V = 426, *p* = 0.002, r = 0.502; Q9: V = 615, *p* < 0.001, r = 0.806; Q10: V = 473, *p* < 0.001, r = 0.745; Q11: V = 520, *p* < 0.001, r = 0.703; Q12: V = 472, *p* < 0.001, r = 0.723; Q13: V = 47.5, *p* = 0.487, r = 0.061. (B) The SCR results. V = 608, *p* = 0.007, r = 0.421. The bold lines indicate the medians; upper and lower limits of the boxplot indicate the 75th and 25th percentiles. The error bars represent 1.5 IQR. Significance levels: ∗*p* < 0.05; ∗∗*p* < 0.01; ∗∗∗*p* < 0.001. (C) CBT results of the synchronous condition. Participants saw five 3D billiard balls with different numbers, appearing one at a time, spaced evenly apart in front of them. From left to right, the numbers were 5, 2, 7, 3, 6. Ball-5 was located to the front-left of the left avatar. Ball-2 was directly in front of the left avatar. Ball-7 was in front of the participant’s visual 1PP. Ball-3 was located directly in front of the right avatar. Ball-6 was to the front-right of the right avatar. A pre-test was conducted before participants stepped onto the Bosu ball. A post-test was carried out after the experiments. (D) CBT results of the asynchronous condition.

Experiment 3 was almost the same as Experiment 2, except that participants’ visual 1PP was set in the middle, but about 60 cm behind the two avatars (Figure 5). The box charts of each question and SCR values are shown in Figures 6A and 6B. The CBT showed that most participants felt as if they were at the location of the adopted visual 1PP (Figures 6C and 6D). The results showed significant differences between the synchronous and asynchronous manipulations on all questions except for the control question: Q1 (double Body ownership) (V = 648, *p* < 0.001, r = 0.728), Q2 (double Proprioceptive-vestibular referral) (V = 357, *p* < 0.001, r = 0.541), Q3 (double Agency) (V = 662, *p* < 0.001, r = 0.841), Q4 (right Body ownership) (V = 513, *p* < 0.001, r = 0.773), Q5 (right Proprioceptive-vestibular referral) (V = 414, *p* = 0.001, r = 0.521), Q6 (right Agency) (V = 777, *p* < 0.001, r = 0.784), Q7 (left Body ownership) (V = 598, *p* < 0.001, r = 0.761), Q8 (left Proprioceptive-vestibular referral) (V = 426, *p* = 0.002, r = 0.502), Q9 (left Agency) (V = 615, *p* < 0.001, r = 0.806), Q10 (right Body-location) (V = 473, *p* < 0.001, r = 0.745), Q11 (left Body-location) (V = 520, *p* < 0.001, r = 0.703), Q12 (double Body-location) (V = 472, *p* < 0.001, r = 0.723), Q13 (Control) (V = 47.5, *p* = 0.487, r = 0.061), and SCR (V = 608, *p* = 0.007, r = 0.421). These results suggest that in the synchronous condition, participants experienced double body ownership and double body-location from this 3PP set-up.

### Experiment 4: 1PP-switch, double body conditions, Sync. vs. Async

**Figure 7:**
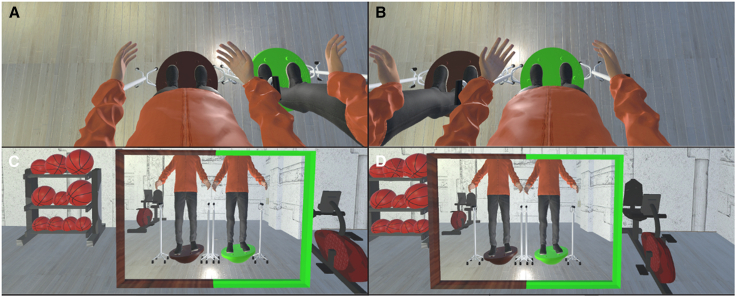
The set-up of Experiment 4 Participants’ 1PP switched back and forth between the two avatars every 20 s throughout the experiment. When they looked down, they saw either (A) the left avatar from the 1PP or (B) the right avatar from the 1PP. When they looked straight ahead, they saw either (C) the left avatar in the virtual mirror directly in front of them, or (D) the right avatar in the virtual mirror directly in front of them.

**Figure 8:**
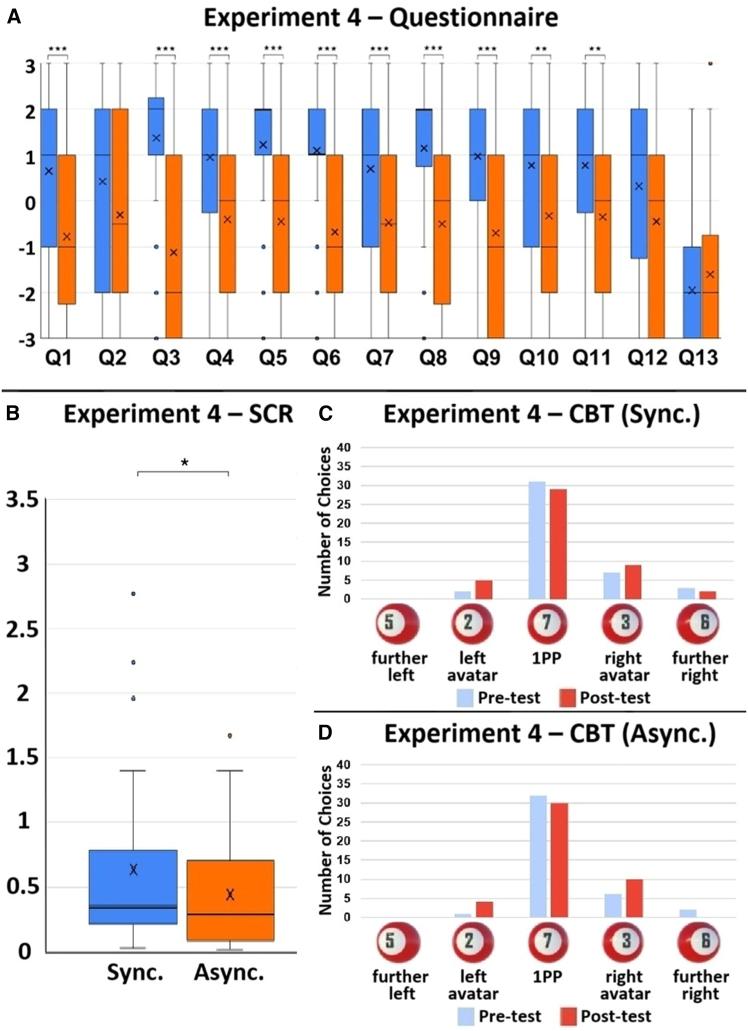
The results of Experiment 4. *N* = 40 (A) The Wilcoxon signed-rank test results of each questionnaire item. Q1: V = 415, *p* = 0.001, r = 0.522; Q2: V = 332, *p* = 0.100, r = 0.286; Q3: V = 640, *p* < 0.001, r = 0.783; Q4: V = 416, *p* = 0.001, r = 0.545; Q5: V = 420, *p* < 0.001, r = 0.709; Q6: V = 483, *p* < 0.001, r = 0.675; Q7: V = 398, *p* < 0.001,r = 0.663; Q8: V = 409, *p* < 0.001, r = 0.662; Q9: V = 442, *p* < 0.001, r = 0.708; Q10: V = 362, *p* = 0.008, r = 0.494; Q11: V = 392, *p* = 0.004, r = 0.476; Q12: V = 270, *p* = 0.052, r = 0.331; Q13: V = 294, *p* = 0.366, r = 0.167. (B) The SCR results. V = 557, *p* = 0.048, r = 0.312. The bold lines indicate the medians; upper and lower limits of the boxplot indicate the 75th and 25th percentiles. The error bars represent 1.5 IQR. Significance levels: ∗*p* < 0.05; ∗∗*p* < 0.01; ∗∗∗*p* < 0.001. (C) CBT results of the synchronous condition. Participants saw five 3D billiard balls with different numbers, appearing one at a time, spaced evenly apart in front of them. From left to right, the numbers were 5, 2, 7, 3, 6. Ball-5 was located to the front-left of the left avatar. Ball-2 was directly in front of the left avatar. Ball-7 was in front of the participant’s visual 1PP. Ball-3 was located directly in front of the right avatar. Ball-6 was to the front-right of the right avatar. A pre-test was conducted before participants stepped onto the Bosu ball. A post-test was carried out after the experiments. (D) CBT results of the asynchronous condition.

The settings of Experiment 4 were almost the same as those of Experiment 2, except that participants’ 1PP switched back and forth between the two avatars every 20 s throughout the experiment (Figures 7A–7D). The box charts of each question and SCR values are shown in Figures 8A and 8B. The CBT showed that most participants felt as if they were located at the place of the adopted visual 1PP (Figures 8C and 8D). Except for Q2, Q12 and Q13, all the other questionnaire data showed significant differences between the synchronous and asynchronous conditions: Q1 (double Body ownership) (V = 415, *p* = 0.001, r = 0.522), Q2 (double Proprioceptive-vestibular referral) (V = 332, *p* = 0.100, r = 0.286), Q3 (double Agency) (V = 640, *p* < 0.001, r = 0.783), Q4 (right Body ownership) (V = 416, *p* = 0.001, r = 0.545), Q5 (right Proprioceptive-vestibular referral) (V = 420, *p* < 0.001, r = 0.709), Q6 (right Agency) (V = 483, *p* < 0.001, r = 0.675), Q7 (left Body ownership) (V = 398, *p* < 0.001, r = 0.663), Q8 (left Proprioceptive-vestibular referral) (V = 409, *p* < 0.001, r = 0.662), Q9 (left Agency) (V = 442, *p* < 0.001, r = 0.708), Q10 (right Body-location) (V = 362, *p* = 0.008, r = 0.494), Q11 (left Body-location) (V = 392, *p* = 0.004, r = 0.476), Q12 (double Body-location) (V = 270, *p* = 0.052, r = 0.331), Q13 (Control) (V = 294, *p* = 0.366, r = 0.167), and SCR (V = 557, *p* = 0.048, r = 0.312). These results suggest that participants experienced body ownership of both avatars even when their 1PP switched.

### Experiment 5: 1PP, double body conditions, scr only

**Figure 9:**
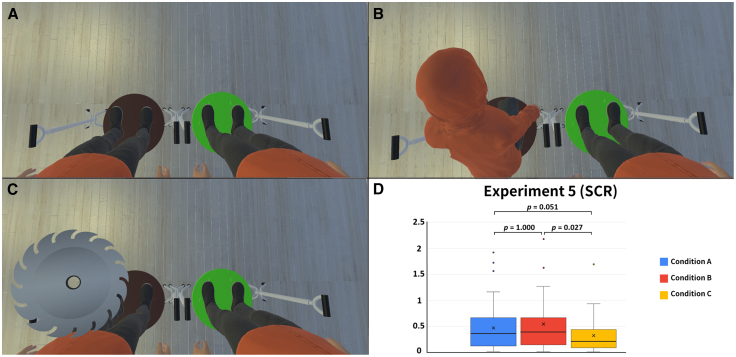
The set-up and results of Experiment 5. *N* = 36 (A) In Condition A, both avatars were synchronous with respect to the participants. When the participants looked down, they saw two life-sized avatars standing on virtual Bosu balls of different colors, and their visual 1PP was located in the middle of the two avatars. (B) In Conditions B and C, one of the avatars was synchronous while the other squatted down and was asynchronous with respect to the participants (randomly selected and evenly distributed). (C) In Conditions A ∼ C, the SCR was measured after a virtual circular saw blade flew toward one of the avatars. (D) The SCR results of each condition. Friedman test: χ^2^ = 9.389, *p* = 0.009, W = 0.130. Post hoc analysis (α adjusted): Condition A vs. Condition B (V = 335, *p* = 1.000, r = 0.005); Condition B vs. Condition C (V = 497, *p* = 0.027, r = 0.429); Condition A vs. Condition C (V = 485.5, *p* = 0.051, r = 0.399). The bold lines indicate the medians; upper and lower limits of the boxplot indicate the 75th and 25th percentiles. The error bars represent 1.5 IQR. Significance levels: ∗*p* < 0.05; ∗∗*p* < 0.01; ∗∗∗*p* < 0.001.

The goal of Experiment 5 was to rule out the possibility that the results of Experiments 2–4 only demonstrated a single-body effect that alternated between the two avatars. The setting was very similar to the synchronous condition of Experiment 2, except that we performed only three rounds of SCR measurements. Condition A: both avatars were synchronous with respect to the participants (Figure 9A), but only one avatar was threatened (randomly selected but evenly distributed) (Figure 9C). Condition B: one avatar was synchronous while the other was asynchronous with respect to the participants (randomly selected but evenly distributed) (Figure 9B), and only the synchronous avatar was threatened (Figure 9C). Condition C: the manipulation was the same as B, but only the asynchronous avatar was threatened. The outcome of the Friedman test was significant among the SCR data (χ^2^ = 9.389, *p* = 0.009, W = 0.130). The post hoc analysis (α adjusted) showed that there was no significant difference between Condition A and Condition B (V = 335, *p* = 1.000, r = 0.005). This suggests that randomly threatening either avatar was the same type of case as threatening only the synchronous avatar. Condition B was significantly higher than Condition C (V = 497, *p* = 0.027, r = 0.429), and Condition A was nearly significantly higher than Condition C (V = 485.5, *p* = 0.051, r = 0.399) (Figure 9D). These data provide physiological evidence supporting that the Double Body Effect is a genuine effect rather than just an alternating single body phenomenon.^26^

### Cross-experiment analyses

We conducted two sets of 3-way mixed-model ANOVAs to analyze whether different manipulations in our experiments influenced participants’ experiences. Since the data did not pass the Shapiro-Wilk test, all ANOVAs were conducted after the Aligned Ranked Transform procedure.

### First set: Perspective vs. synchronicity across experiential dimensions

We conducted three 3-way mixed-model ANOVAs to test whether the perspectival difference between 1PP and 3PP and/or synchronicity resulted in different experiences with regard to the right, the left, and both avatars, respectively. The first ANOVA targeted the experiences of both avatars. The factors were 2 [Perspectival Difference: 1PP (Experiment 2) vs. 3PP (Experiment 3)] × 2 [Synchronicity: synchronous conditions vs. asynchronous conditions] × 4 [Double Body Experience Items (DBEI): body ownership (Q1) vs. proprioceptive-vestibular referral (Q2) vs. agency (Q3) vs. body-location (Q12)]. A main effect was observed in Synchronicity (*F*_(1,546)_ = 285.326, *p* < 0.001, *η*_*p*_^2^(ART) = 0.343) and DBEI (*F*_(3,546)_ = 8.232, *p* < 0.001, *η*_*p*_^2^(ART) = 0.043). Interaction effects were found in Perspectival Difference × Synchronicity (*F*_(1,546)_ = 4.163, *p* = 0.042, *η*_*p*_^2^(ART) = 0.008) and in Synchronicity × DBEI (*F*_(3,546)_ = 7.763, *p* < 0.001, *η*_*p*_^2^(ART) = 0.041). The post-hoc analyses revealed that the interaction effects stemmed from the significant differences between the synchronous and asynchronous conditions across different perspectives and from the relatively high proprioceptive-vestibular referral ratings (Q2) in the asynchronous conditions.

The second ANOVA was about the experiences of the right avatar. The factors were 2 [Perspectival Difference: 1PP (Experiment 2) vs. 3PP (Experiment 3)] × 2 [Synchronicity: synchronous conditions vs. asynchronous conditions] × 4 [Right Body Experience Items (RBEI): body ownership (Q4) vs. proprioceptive-vestibular referral (Q5) vs. agency (Q6) vs. body-location (Q10)]. Main effects were observed in all factors: Perspectival Difference (*F*_(1,78)_ = 5.737, *p* = 0.019, *η*_*p*_^2^(ART) = 0.069), Synchronicity (*F*_(1,546)_ = 375.184, *p* < 0.001, *η*_*p*_^2^(ART) = 0.407), and RBEI (*F*_(3,546)_ = 8.421, *p* < 0.001, *η*_*p*_^2^(ART) = 0.044). An interaction effect was found in Synchronicity × RBEI (*F*_(3,546)_ = 7.334, *p* < 0.001, *η*_*p*_^2^(ART) = 0.039). The post-hoc analyses indicated that these effects were due to the significant differences between the synchronous and asynchronous conditions across different perspectives and the relatively high proprioceptive-vestibular referral ratings (Q5) in the asynchronous conditions.

The third ANOVA targeted the experiences of the left avatar. The factors were 2 [Perspectival Difference: 1PP (Experiment 2) vs. 3PP (Experiment 3)] × 2 [Synchronicity: Synchronous conditions vs. Asynchronous conditions] × 4 [Left Body Experience Items (LBEI): body ownership (Q7) vs. proprioceptive-vestibular referral (Q8) vs. agency (Q9) vs. body-location (Q11)]. Main effects were observed in Synchronicity (*F*_(1,546)_ = 351.586, *p* < 0.001, *η*_*p*_^2^(ART) = 0.392) and LBEI (*F*_(3,546)_ = 4.017, *p* = 0.008, *η*_*p*_^2^(ART) = 0.022). An interaction effect was observed in Synchronicity × LBEI (*F*_(3,546)_ = 5.644, *p* = 0.001, *η*_*p*_^2^(ART) = 0.030). The post-hoc analyses revealed that these effects stemmed from the significant differences between the synchronous and asynchronous conditions across different perspectives and from the relatively high proprioceptive-vestibular referral ratings (Q8) in the asynchronous conditions.

### Second set: Perspective vs. synchronicity within experiential dimensions

We conducted another five 3-way mixed-model ANOVAs to test whether the perspectival difference between 1PP and 3PP and/or synchronicity influenced body ownership, proprioceptive-vestibular referral, agency, and body location, respectively. The first ANOVA targeted body ownership. The factors were 3 [Perspectival Difference: 1PP (Experiment 2) vs. 3PP (Experiment 3) vs. 1PP-Switch (Experiment 4)] × 2 [Synchronicity: synchronous conditions vs. asynchronous conditions] × 3 [Body Ownership Items: both avatars (Q1) vs. right avatar (Q4) vs. left avatar (Q7)]. A main effect was observed in Synchronicity (*F*_(1,585)_ = 378.374, *p* < 0.001, *η*_*p*_^2^(ART) = 0.393). An interaction effect was observed for Perspectival Difference × Synchronicity (*F*_(2,585)_ = 7.099, *p* = 0.001, *η*_*p*_^2^(ART) = 0.024). The post-hoc analyses revealed that the interaction effect stemmed from the significant differences between the synchronous and asynchronous conditions across different perspectives.

The second ANOVA was about proprioceptive-vestibular referral. The factors were 3 [Perspectival Difference: 1PP (Experiment 2) vs. 3PP (Experiment 3) vs. 1PP-Switch (Experiment 4)] × 2 [Synchronicity: synchronous conditions vs. asynchronous conditions] × 3 [Referral Items: both avatars (Q2) vs. right avatar (Q5) vs. left avatar (Q8)]. A main effect was observed for Synchronicity (*F*_(1,585)_ = 173.182, *p* < 0.001, *η*_*p*_^2^(ART) = 0.228). No interaction effect was found.

The third ANOVA targeted agency. The factors were 3 [Perspectival Difference: 1PP (Experiment 2) vs. 3PP (Experiment 3) vs. 1PP-Switch (Experiment 4)] × 2 [Synchronicity: synchronous conditions vs. asynchronous conditions] × 3 [Agency Items: both avatars (Q3) vs. right avatar (Q6) vs. left avatar (Q9)]. A main effect was observed for Synchronicity (*F*_(1,585)_ = 662.898, *p* < 0.001, *η*_*p*_^2^(ART) = 0.537). An interaction effect was observed for Perspectival Difference × Synchronicity (*F*_(2,585)_ = 5.184, *p* = 0.006, *η*_*p*_^2^(ART) = 0.017). The post-hoc analyses showed that the interaction effect was due to the significant differences between the synchronous and asynchronous conditions across different perspectives.

The fourth ANOVA concerned body location. The factors were 3 [Perspectival Difference: 1PP (Experiment 2) vs. 3PP (Experiment 3) vs. 1PP-Switch (Experiment 4)] × 2 [Synchronicity: synchronous conditions vs. asynchronous conditions] × 3 [Body-location Items: both avatars (Q12) vs. right avatar (Q10) vs. left avatar (Q11)]. Main effects were observed for Perspectival Difference (*F*_(2,117)_ = 3.358, *p* = 0.038, *η*_*p*_^2^(ART) = 0.054) and Synchronicity (*F*_(1,585)_ = 212.848, *p* < 0.001, *η*_*p*_^2^(ART) = 0.267). An interaction effect was found for Perspectival Difference × Synchronicity (*F*_(2,585)_ = 6.886, *p* = 0.001, *η*_*p*_^2^(ART) = 0.023). The post-hoc analyses revealed that these effects stemmed from the significant differences between 1PP (Experiment 2) and 3PP (Experiment 3) (*p* = 0.048, Bonferroni-corrected α value) and between the synchronous and asynchronous conditions across different perspectives.

Finally, the fifth ANOVA considered all the above dimensions together. The factors were 3 [Perspectival Difference: 1PP (Experiment 2) vs. 3PP (Experiment 3) vs. 1PP-Switch (Experiment 4)] × 2 [Synchronicity: synchronous conditions vs. asynchronous conditions] × 3 [both avatars (Q1+Q2+Q3+Q12) vs. right avatar (Q4+Q5+Q6+Q10) vs. left avatar (Q7+Q8+Q9+Q11)]. A main effect was observed for Synchronicity (*F*_(1,585)_ = 518.154, *p* < 0.001, *η*_*p*_^2^(ART) = 0.469). An interaction effect was found for Perspectival Difference × Synchronicity (*F*_(2,585)_ = 4.291, *p* = 0.014, *η*_*p*_^2^(ART) = 0.014). The post-hoc analyses showed that the interaction effect was due to the significant differences between the synchronous and asynchronous conditions across different perspectives.

## Discussion

In this study, we applied VR technologies and integrated proprioceptive-vestibular signals with visual information to investigate bodily self-consciousness (BSC). In Experiment 2, the 1PP conditions, our results showed that participants felt from the 1PP as if both avatars were their own bodies, and their sense of body-location was felt to be at the places of both avatars. In Experiment 3, similar experiences of double body ownership and double body-location were induced even from the 3PP. In Experiment 4, the sense of double body ownership was induced by the 1PP-swich setting. With regard to double body-location, the result of the synchronous condition was nearly significantly higher than the asynchronous condition, supported by significant results of both left and right body-location. This suggests that the experience of double body-location remains possible in the 1PP-swich setting. Finally, the SCR data of Experiment 5 exclude a potential confounding interpretation. Together, we demonstrate that it is empirically possible for healthy subjects to experience the Double Body Effect.

Our experiments involved two kinds of movements: active and passive. In the synchronous conditions of Experiments 1–4, the participants were instructed to perform active movements, including moving their body freely, looking down, and swinging their arms. We do not think that it was these active movements that engendered the Double Body Effect because this effect is unusual and very hard to induce, and is certainly and not easily triggered by visual-motor congruence alone. Active movements were also involved in the asynchronous conditions to accentuate the mismatch between movements and visual feedback (cf. STAR Methods). In any case, active movements were not the main manipulations in this study. We think that the Double Body Effect was mainly due to the passive movements and their correlations with visual feedback. Our experiments were distinct from many previous studies in that we used a Bosu ball to trigger participants’ proprioception and their vestibular system, and that the participants’ wobbling movement on the Bosu ball was mostly involuntary. The key factor for inducing the Double Body Effect, we believe, was the synchronous match between visual feedback and involuntary wobbling. In the following, we compare our experiments with some previous studies, then discuss the implications of the present study.

(1) In Heydrich et al.,^27^ two different methods (one using an HMD-camera set-up and the other using VR techniques) were applied to create identical images of two bodies. Participants watched two virtual bodies about 2 m in front of them from the 3PP. They received tactile stimulation on the back while watching the two virtual bodies being stroked in the corresponding areas. Unfortunately, the measurement of double body ownership solely relied on questionnaires, without the support of physiological evidence such as SCR. Also, the median ratings of a key questionnaire statement (“It seemed as if I might have more than one body”) were not robust.
(2) In Aymerich-Franch et al.,^64^ participants controlled a humanoid robot with a joystick, wore an HMD that adopted the viewpoint of the robot, and saw their own body from the perspective of the robot. Although some of questionnaire data suggested that “healthy humans can bi-locate in two different bodies at the same time,”^64^ the questionnaires did not contain statements regarding double body ownership. Also, the authors did not perform either behavioral or SCR measurements to support their data.

In comparison, while Heydrich et al.^27^ used only 3PP set-ups and Aymerich-Franch et al.^64^ included only 1PP condition, the present study included both the 1PP and 3PP conditions. Heydrich et al.^27^ used the usual visual-tactile stimulations; in contrast, our manipulations integrated proprioceptive-vestibular and visual information. Both Aymerich-Franch et al.^64^ and our study involved the sense of agency, although in different ways. Moreover, our experimental designs avoided the drawbacks of these two studies. The questionnaires in our study contained specific statements about double body ownership and double body-location with several supporting statements, and the data were backed by SCR evidence. The results that we observed were more robust than these two earlier works, and hence provided better grounds for the Double Body Effect.

(3) In Guterstam et al.,^26^ participants wore an HMD, lay in bed, and watched two identical bodies situated in the same postures from the 1PP. The sense of owning two bodies was observed under synchronous visual-tactile stimulations, supported by SCR evidence. When participants switched perspectives between 1PP and 3PP, it was reported that both dual body ownership and dual self-location were observed in the synchronous 1PP-1PP and the synchronous 1PP-1PP-3PP conditions. Further support was provided by the results of a self-location task: a map was presented on the HMD that marked the location of the participant’s real body and/or the locations of the two illusory bodies. Participants rated how strongly they felt that they were located in those places. Dual self-location was rated higher in the synchronous 1PP-1PP and the 1PP-1PP-3PP conditions than in the 3PP-only condition. Some of the data were a bit weak: In their perspective-switching experiment, the absolute ratings of the key dual body ownership statement (S4) in both the synchronous 1PP-1PP (rating = 0.1) and the synchronous 1PP-1PP-3PP (rating = 0.3) conditions were rather low. Regarding all ownership-related statements (S4 ∼ S6), there were no significant differences between the synchronous 1PP-1PP and the synchronous 1PP-1PP-3PP conditions, on the one hand, and the synchronous 3PP-only condition, on the other. Notwithstanding these minor results, the ratings of the key dual body ownership statements (S1 ∼ S2) in the main experiment (Experiment 1) were robust under synchronous manipulations (mean = 1.4) and were significantly higher than the asynchronous condition.^26^ We think that this study by Guterstam et al.^26^ was more successful than the two studies mentioned above.

In comparison, instead of visual-tactile stimulations, we manipulated proprioceptive-vestibular and visual signals. Our experiments replicated dual body ownership both from the 1PP and from the 3PP. It was not clear whether Guterstam et al.^26^ distinguished between self-location and body-location. We used different methods to measure self-location (CBT) and body-location (questionnaires). Our data suggest that they are different phenomena. We also suggest that another factor—1PP-location—should be taken into consideration when it comes to understanding self-location. See the discussion later in discussion on the implications of the present study.

(4) In Preuss and Ehrsson,^48^ participants saw an avatar lying on a bed in a virtual room from the 1PP, and received galvanic vestibular stimulation (GVS). The avatar appeared to be slowly swinging (6°/s oscillations, from left to right, back and forth) and GVS induced swinging-like motion sensations. When the visual stimulations and the GVS-induced sensations were synchronous and in the same direction, questionnaire data suggested that the participants experienced illusory ownership of the avatar (cf. another study using GVS by Preuss Mattsson et al.^49^). The design of this study was limited to single body ownership and the 1PP set-up. In comparison, the current study substantially extends the flexibility of body ownership to double bodies and successfully induced the Double Body Effect using both a 1PP set-up (Experiment 2) and a 3PP set-up (Experiment 3). Also, as indicated by Preuss and Ehrsson,^48^ there was a limitation of the GVS method; that is, since it was hard to control the exact onset of the vestibular sensations, it was difficult to make sure that the visual and vestibular stimulations were fully synchronized in the congruent conditions. In our study, this problem of synchronization was solved by attaching motion trackers to the participants and to the Bosu ball. Then, careful VR programming allowed us to create perfect correlations between participants’ real wobbling movements and what they saw in the virtual gym.
(5) Wu et al.^50^ used a 3PP set-up in which participants lay on a Stewart-Gough motion platform and saw a 3D reconstruction of their body from the 3PP in a mixed reality environment. The motion platform moved the participants’ whole bodies up or down to provide vestibular stimulation. The visual manipulations consisted of rotating the participants’ viewpoint 180°, then looking downward at their virtual body from a short distance. In the congruent condition, these integrated visual-vestibular stimulations generated OBE-like sensations, that is, the sense of elevated self-location and the subjective feelings of disembodiment and lightness. Like Preuss and Ehrsson,^48^ the design of Wu et al.^50^ focused on single body ownership, but it was limited to the 3PP set-up. Our current study makes progress by aiming at the Double Body Effect using both the 1PP and 3PP set-ups. Also, Wu et al.^50^ pointed out that their results did not show significant differences between congruent and incongruent conditions with regard to full-body ownership. They proposed that “vestibular information and visual-vestibular integration may be more important for dis/embodiment and self-location than for self-identification and body ownership.”^50^ In comparison, we incorporated proprioception into manipulations in addition to visual-vestibular correlation. Our study suggests that the integration of proprioceptive-vestibular and visual information has a great influence on the Double Body Effect. Such an integration is certainly as important for full-body ownership as for self-location.

To conclude this part of the discussion, our study provides a useful approach at the behavioral and psychophysical levels for investigating BSC. Many neuroscientific studies have suggested that BSC essentially depends on integration between exteroceptive and environmental information, on the one hand, and proprioceptive-vestibular and interoceptive information, on the other.^7^^,^^14^^,^^16^^,^^41^^,^^42^^,^^44^ By integrating visual and proprioceptive-vestibular information, our approach can help connect the malleable phenomenological features of BSC and the research of its neural mechanisms.

How do the findings of the present study enhance the current understanding of BSC? First, our results show that the sense of body ownership and the sense of body location are, in fact, much more flexible than most researchers have suggested. For example, based on a set of experiments that reported “disownership” of participants’ real body, Guterstam and Ehrsson^65^ remarked that “it might not be possible for a healthy brain to perceive the self to be located at two different places at the same time and owning two different bodies at these locations.” However, this view is seriously challenged by our data on the Double Body Effect. It is possible for healthy subjects to experience not only ownership of extra limbs^24^^,^^25^^,^^30^ but also ownership of two full bodies, as well as two separate body-locations at the same time.

Second, our findings recommend a cautious and critical stance on some influential views of BSC. When searching for the neural mechanisms for BSC, many researchers turn their attention to brain areas containing bimodal or multimodal neurons that encode multisensory stimuli, such as visual, somatosensory, proprioceptive, and vestibular signals, within the limb-centered or trunk-centered peripersonal space.^5^^,^^14^^,^^28^^,^^34^^,^^54^^,^^56^ The idea is that those brain areas (and connectivity among them) may be where multisensory bodily signals are integrated, and it is such integration that gives rise to BSC. Although this idea is widely accepted, the difficult task is to ascertain exactly how it works. It can be developed in different ways^19^^,^^28^^,^^29^^,^^44^; here we discuss an important one. In a series of articles, Blanke and colleagues proposed to explain body ownership in terms of enlargement and relocation of receptive fields of bimodal/multimodal neurons in certain brain areas such as the premotor cortex (PMC), intraparietal sulcus (IPS), and insula.^5^^,^^10^^,^^42^ This explanation intends to cover not only limb ownership but also full-body ownership: “On the basis of the involvement of the IPS and PMC in humans in both hand ownership and self-identification (that is, body ownership) and the properties of bimodal visuotactile neurons in these regions in monkeys, it can be speculated that changes in full-body self-identification may be a result of stroking-induced changes in the size and position of trunk-centered bimodal neurons with respect to the virtual body that is seen on the HMD. In this scenario, the visual receptive fields of such bimodal neurons would be enlarged following visuotactile stroking, and would also encode the more distant position of the seen virtual body after stroking.”^10^

Now, in light of the data on the Double Body Effect, we have reservations about this neurophysiological explanation with regard to full-body ownership. While there is some evidence supporting this explanation in the case of body-part ownership, whether (illusory) full-body ownership is due to the malleability of receptive fields of bimodal/multimodal neurons still awaits confirmation. As Blanke stated, “no direct evidence for this possibility exists yet.”^10^ More importantly, evidence for the case of double body ownership and double body-location is currently lacking. From our perspective, the challenge is as follows: since the Double Body Effect is empirically possible, in order for this explanation to work it seems to require evidence showing that the changes in the size and location of the receptive fields of relevant multimodal neurons are in fact huge enough to encompass the scope of double bodies in separate locations, and that the coding processes that use the trunk-centered reference frame in the PMC-IPS-insula network can accommodate the phenomenology of the Double Body Effect. This clearly exceeds any current data that might support this explanation. Future research in this direction should take the Double Body Effect into consideration.

Third, another implication of our experiments concerns the distinction between 1PP and 3PP in BSC. In the study of the rubber hand illusion and full-body illusions, many researchers regard visual 1PP as a fundamental constraint of body ownership.^19^^,^^28^^,^^34^^,^^66^ For example, Petkova et al.^34^ claimed that “the first person visual perspective would represent a fundamental constraint on the full-body illusion.” They further contended that “the first person visual perspective is critical for triggering the illusion of full-body ownership. This is an important observation as it shows that the very basic sensation of owning one’s body is the result of a constructive process where visual, tactile, and proprioceptive signals are integrated in ego-centric coordinates.” According to this view, a bodily illusion induced by a 3PP-setup (i.e., the illusory body seen from a distance as if one is looking at someone else’s body; e.g., Lenggenhager et al.^32^) is regarded as merely a result of visual self-recognition rather than a “genuine body illusion.”^28^^,^^34^ As we see it, there is an underlying assumption behind this view, that is, the distinction between 1PP and 3PP is rigid, such that experiences based on 1PP are fundamentally different from experiences based on 3PP. This assumption has support from several studies.^34^^,^^55^^,^^66^^,^^67^^,^^68^ However, some other studies, including the present one, raise the question: Is this assumption correct? Let us briefly consider two sets of studies.

In the fMRI study by Petkova et al.,^67^ a 1PP condition (an artificial body seen from the visual 1PP in near-personal space) was compared with a 3PP condition (the artificial body seen from the visual 3PP in far extrapersonal space). Significantly stronger activities were recorded in the right PMv, the left IPS, and the left putamen in the synchronous 1PP condition. Using visual-tactile stimulations, Petkova et al.^34^ showed that the body ownership illusion induced in the 1PP condition was significantly stronger than that induced in the 3PP condition (a mannequin viewed as facing the participants from a distance of 75 cm). Petkova et al.^34^ also showed that the illusion can occur from the 1PP without using an HMD, which was not the case from the 3PP (where the mannequin was presented in the opposite direction and 1 m apart to the right). Similar results were observed in a VR study by Maselli and Slater,^66^ where the virtual body in the 3PP condition was seen 40 cm away to the left. The authors concluded that “first person perspective over the fake body is a necessary condition for the onset of the full body ownership illusion” and that the “violation of 1PP over the fake body is sufficient to prevent the full body ownership illusion, even if the body is seen in the peripersonal space.”^66^ In another study, Maselli and Slater^55^ investigated full-body ownership and self-location in three different conditions: 1PP-TO (viewing a virtual body from the 1PP with total overlap between the real and virtual bodies), 3PP-PO (viewing a virtual body from the 3PP with partial overlap between the real and virtual bodies), 3PP-NO (viewing a virtual body from the 3PP with no overlap between the two bodies). Results showed that the full-body ownership illusion occurred only in 1PP-TO but not in 3PP-NO. Finally, in the VR study by Gorisse et al.,^68^ the questionnaire data indicated that “the 1 PP condition is significantly superior to the 3PP condition.” These studies showed that, given the specific set-ups, the illusory experience of body ownership was significantly weaker when induced from the 3PP, and hence provided support for the assumption mentioned above. So, the question is: Is the support strong enough to establish that visual 1PP is a “fundamental constraint” such that genuine body ownership illusions can *only* be induced from the 1PP and *not* by 3PP setups? If the answer is yes, then one can say that experiences based on 1PP are indeed fundamentally different from experiences based on 3PP.

However, there is another set of studies that suggest otherwise. In Maselli and Slater,^55^ just described above, there were no significant differences between 1PP-TO and 3PP-PO on several questionnaire statements regarding body ownership. As the authors said, “a strong FBOI [full-body ownership illusion] occurred similarly in both 1PP-TO and 3PP-PO.” Preston et al.^69^ showed that viewing a mannequin through a mirror (i.e., from the 3PP) plus synchronous tactile stimulations could elicit illusory body ownership over the mannequin. The authors suggested that the visual information from the mirror reflection “was referred back to the peripersonal space around the participant’s own body” and “combined with tactile and proprioceptive information according to an ego-centric spatial reference frame.” Such a 3PP full-body illusion via a mirror was “statistically equivalent” to that induced from the 1PP.^69^ In the VR study by Galvan Debarba et al.,^53^ participants controlled and watched an avatar either from the 1PP or from the 3PP (120 cm in the front) and received haptic feedback on their feet. The data of the questionnaire and galvanic skin response (GSR) suggested that “illusory ownership of a virtual body can be achieved in both first and third person perspectives under congruent visual-motor-tactile conditions.” Bourdin et al.^70^ used a 3PP set-up to induce what they called “Drifting Body Experience” in a virtual environment. After an initial phase of receiving visual-vibrotactile stimulations from the 1PP, participants’ viewpoint was lifted up to the ceiling. They watched from above an avatar being stroked by virtual balls, and at the same time received corresponding vibrotactile stimulations. Results indicated that the participants “tended to affirm body ownership and connection with the virtual body that they saw below.” In the VR study by Liou et al.,^71^ participants were immersed in a virtual room and watched a life-sized avatar either from the 1PP or from the 3PP (the avatar appeared at the 45° right-front side about 45 cm away). In two 3PP experiments, when the participants looked down at their body, they would see nothing there; rather, they would have to look to the right front about 45° in order to see the avatar. The participants actively interacted with a virtual soccer ball and/or passively received tactile stimulations. Questionnaire and SCR data showed that, by using visual-motor-tactile and visual-tactile correlations, the sense of full-body ownership over the avatar and the sense of body-location can be induced not only in the 1PP condition but also in the active and the passive 3PP conditions. Finally, in the current study, we have shown that the Double Body Effect can be experienced not only from the 1PP (Experiment 2) but also from the 3PP (Experiment 3). Together, these studies show that genuine bodily illusions can be engendered by at least some 3PP set-ups. This in turn suggests that, with regard to full-body ownership and body-location, the differences between 1PP-experience and 3PP-experience are at most a matter of degree.

We do not think that the empirical results of the above two sets of studies really conflict with each other. They are only interpreted as tending in different directions. Notice that the 3PP conditions in the first set of studies and the 3PP conditions in the second set were all different. The key is that, although in *some* experimental settings the illusory sense of body ownership was significantly weaker from the 3PP than from the 1PP, that does *not* automatically prevent genuine body ownership illusions from being generated by other 3PP experiments. Therefore, given the studies that induced bodily illusions from the 3PP, we think that the assumption mentioned above does not hold. The distinction between embodied 1PP and embodied 3PP is *not* rigid, but is at most a matter of degree. Once the assumption is dropped, we can see that the data of those studies that emphasized the differences between 1PP- and 3PP-experiences, on the one hand, and those studies suggesting that bodily illusions can be induced from the 3PP, on the other, are actually compatible with each other. It is just that the distinction between 1PP- and 3PP-experiences is a matter of degree.

Notice that in all experiments of the present study, including Experiment 3, the participants’ 1PP played an important role. What made Experiment 3 special was that we moved the participants’ 1PP-location 60 cm behind, such that the two avatars were seen from a short distance, that is, from the 3PP. The induced Double Body Effect was causally related to the participants’ 1PP because the effect was partially due to the manipulation of their 1PP location. Therefore, in suggesting that the distinction between 1PP- and 3PP-experiences is not rigid, we are not refuting that the body ownership illusion induced from the 1PP is significantly stronger than that from the 3PP in many specific set-ups. We are neither denying that visual 1PP is important for the experience of body ownership, nor are we negating that body ownership requires multisensory signals to be integrated into ego-centric coordinates. However, we do think that the distinction between embodied 1PP and embodied 3PP is subtle and more complicated than most researchers have thought, and that *how* to characterize visual 1PP as a constraint of body ownership needs to be reconsidered.

Fourth, our results suggest that body-location and self-location are distinct phenomena. While the participants in Experiments 2 and 3 experienced double body-location, the CBT results showed that their sense of self-location was not duplicated and was felt at the location of the adopted visual 1PP. There are two reasons why this was the case. The first is methodological. In our experiments, we programmed a single viewpoint in the virtual environment such that the position and orientation of that viewpoint corresponded to the position and orientation of participants’ HMD in the real world. Hence, this viewpoint corresponded to participants’ 1PP-location. Nonetheless, when we conducted the CBT, the participants were specifically informed beforehand that multiple choices were allowed. So, whether the CBT data would accord with the data of body location can only be determined by the experimental results. The second reason concerns the relations between self-location, body-location, and 1PP-location. There are good reasons suggesting that self-location is closely related to 1PP-location, and that 1PP-location and body-location are not the same phenomenon. Let us elaborate.

Many researchers assume that the experience of where I am in space (self-location) is identical to the experience of where my body is located in space (body-location), or at least do not distinguish between them. For example, in the review article by Serino et al.,^53^ self-location was defined as “the experience of being a body with a given location within the environment.” Maselli and Slater^55^ characterized self-location as “the experience of the body occupying a given portion of space in the environment.” In the fMRI study by Guterstam et al.,^54^ self-location was construed as “the experience that the body is located somewhere in space.” In a recent review of heautoscopy by Szczotka and Wierzchoń,^57^ self-location was depicted as “my body has a particular location in space.” However, as mentioned in the introduction, the case of out-of-body experience (OBE) raises doubts about this assumption. During an OBE, subjects have the experience of their self being located outside of their body and watching their body from an external and elevated perspective.^58^ This clearly suggests that during an OBE, the sense of self-location is dissociable from the sense of body location. In contrast, the sense of self-location is closely connected with the sense of 1PP-location. If self-location and body-location were the same, it would be difficult to accurately depict the phenomenology of OBE. More importantly, as reported in the results section, while the experience of double body-location was induced in Experiments 2 and 3, the CBT data strongly suggest that the participants’ sense of self-location in these experiments did *not* split into two. These results suggest that self-location and body-location are not the same phenomena. Rather, it was participants’ sense of 1PP-location that played an important role in their sense of self-location during the experience of the Double Body Effect. In fact, the tight link between self-location and 1PP-location is supported by the fMRI study by Ionta et al.^72^ and others.^10^^,^^42^

Notice that if one has the experience of being outside of one’s own body, that does not imply that one thereby has no bodily awareness at the extrapersonal position at all. For example, in the out-of-body experiments conducted by Lenggenhager et al.,^32^ the authors stated that “our healthy participants did not report feelings of overt disembodiment.” The present study is consistent with this observation. In suggesting that self-location and body-location can sometimes dissociate, we are *not* proposing some sort of Cartesian dualism, according to which self and body are metaphysically distinct. Nor are we suggesting that the self is disembodied. As we see it, the self is essentially embodied,^2^^,^^15^ and the fundamental form of self-consciousness is rooted in bodily experiences. One might wonder: If the self is neither disembodied nor some sort of Cartesian ego, how can self-location be different from body-location? Here is our view: although the self is embodied, this does not imply that self-location and body-location are the same phenomena. The key is that there is another factor, i.e., 1PP-location, which is different from body-location, that bears a close connection with self-location. The CBT results of our Experiments 1–4 showed that participants’ sense of 1PP-location was highly influential in their sense of self-location. This is corroborated by the case of OBE. Also, 1PP-location and body-location are not the same experience, as indicated by the case of OBE, the study by Huang et al.^59^ briefly described in the introduction, and the data of the present study.

If 1PP-location is indeed distinct from body-location, why is this so? Since self-location is closely linked to 1PP-location, clarifying the distinction between 1PP-location and body-location will help us understand why self-location should not be identified with body-location without inquiry. In holding that 1PP-location and body-location are different experiences, we are not suggesting that 1PP-location is an abstract geometric point. There is a sense of embodiment associated with 1PP-location, i.e., we feel that we are embodied in that location, from where we perceive and move around the environment. Like the sense of body-location, the sense of 1PP-location is maintained and influenced by proprioception-vestibular information (leaving interoception aside for the moment). If so, why are 1PP-location and body-location different?

We think that the answer to this question has to do with two things: (i) The ways that proprioception and the vestibular system affect 1PP-location are not the same as the ways they affect body-location. Consider visual 1PP-location, which is mainly regulated by the extraocular muscles and the vestibular-ocular reflex.^73^ In the standard RHI, participants’ head and torso remain stationary. When the illusion occurs, the sense of 1PP-location stays the same while the sense of hand-location changes. While the extraocular muscles and the vestibular-ocular reflex keep participants’ eyes fixated on the fake hand, the drift of the sense of hand-location is caused by visual capture of proprioceptive information of the real hand due to visual-tactile stimulations. The situation is more complicated when 1PP-location and body-location dissociate in some full-body illusions, but it is still the case that body-location and 1PP-location are under (at least partially) different influences of various types of proprioceptive-vestibular information. With regard to body location, it is reasonable to hypothesize that the most important explanatory factor would be the integrated proprioception-vestibular and visual information about the torso. In contrast, the key factor for 1PP-location would be the proprioceptive-vestibular information from the extraocular muscles and the vestibular-ocular reflex. Thus, when 1PP-location and body-location dissociate, the distinction between them can be explained by appealing to such differences. More studies are required to fully articulate the details in specific settings.

(ii) 1PP has some distinctive features that the body does not. The most crucial one is the following (again, take vision as an example): We can see our own hands, legs, and the whole body by simply looking down to our body or by looking in a mirror. When we do that, the body is an object of our visual perception. In contrast, we do not see our own 1PP. Why? Because 1PP is the *origin* of the egocentric spatial framework of our perceptual experiences and movements. We perceive and interact with the environment *from* our 1PP. It is the origin *from which* we see things, thus it itself is not something that we see.^9^^,^^15^ We sense our 1PP with its location and orientation not by vision, but via proprioceptive and vestibular signals. I can see my eyes in a mirror, but I do not thereby see my 1PP. The eyes in the mirror are objects of my visual perception. They do not constitute the 1PP from which I see my body in the mirror. Similarly, in the experiments of the current study, the participants saw the two avatars in the virtual gym from a specific viewpoint that we programmed. But they did not and could not see the viewpoint itself. What does this feature have to do with the distinction between 1PP-location and body-location? Researchers agree that visual capture played an important role in many experiments that induced body-part or full-body illusions.^8^^,^^27^^,^^47^^,^^50^ We think so, too, with regard to the Double Body Effect. For proprioceptive or vestibular information (setting tactile information aside for the moment) to be dominated by vision in the RHI or full-body illusions, participants need to see a fake hand or a virtual body (or bodies) in specific locations. The point is that there needs to be objects of visual perception in order for visual capture to take place. In this regard, since the body can be seen but 1PP cannot, we think that it is likely that 1PP-location is less susceptible to visual capture than is body-location.

There are two other related features of 1PP worth mentioning. One is that each of us is associated with a *unique* 1PP. The 1PP that I have is mine and mine alone. Your 1PP is a 3PP to me, and mine is a 3PP to you. The other feature concerns the sense of location. Wherever I go, my 1PP is always *here* because it is my 1PP that defines and underlies what I feel as being “here.” If you see your eyes in a mirror, that is not *where* your 1PP is, and you will not feel that your 1PP is located in the mirror. It is significant that these features of 1PP can characterize our basic sense of self as well. As mentioned in the Introduction, the most basic sense of self that we have is the sense that each of us is a subject of conscious experience. Each of us is associated with a unique sense of self. Wherever I go, my sense of self is always felt to be *here*. When I see myself in a mirror, my sense of self does not feel as if it is located in the mirror. What I see in the mirror is what philosophers call *self-as-object*, i.e., an object of visual perception, rather than *self-as-subject*, i.e., the subject who is having this visual experience.^6^^,^^9^^,^^74^ Therefore, as we suggest in the Introduction, to be a subject is to have a 1PP as the origin and anchor of the ego-centric reference frame that structures one’s conscious experiences. To fully articulate the distinctive features of 1PP would require further interdisciplinary investigations, but we think that our remarks above can provide at least an initial and plausible explanation of why 1PP-location is different from body-location and why self-location and body-location are distinct phenomena.

Finally, our study shows that the Double Body Effect can serve as a useful preliminary model for understanding the puzzling phenomenology of heautoscopy. As mentioned in the Introduction, patients with heautoscopy have visual perceptions of a *doppelgänger* in the extrapersonal space, and more strangely, they may have the experiences of “bilocation” and of “perspective-switching.”^1^^,^^16^ Although some patients underwent heautoscopy during a complex partial seizure^60^^,^^61^ and heautoscopy was suggested to be related to vestibular disturbance and other defective multisensory integration of bodily signals^10^^,^^58^^,^^62^ and related to “left temporoparietal lesions including the TPJ,”^63^ it has long been difficult to really comprehend this weird syndrome because some patients may undergo similar neurological conditions but have different experiences such as OBE rather than heautoscopy. Also, many case studies and neuroimaging studies suffered from “mislabeling much more common autoscopy or out-of-body experience as heautoscopy” and/or “conceptual and terminological contradictions.”^57^ Thus, how do we make progress?

Before a suitable *explanans* can be offered, we need a more precise depiction of the *explanandum*. The first step should be to unpack and disentangle the complex phenomenology of heautoscopy in an empirically reliable way. For example, regarding “bilocation,” Szczotka and Wierzchoń^57^ maintained that researchers should “strive to disambiguate if the symptoms involve a rapid shift in self-location or rather a sense of occupying two positions at once.” We fully agree with this remark. However, we disagree with their construal of self-location solely in terms of body-location. Furthermore, Szczotka and Wierzchoń^57^ characterized the distinct features of heautoscopy as “a collection of symptoms encompassing an ambiguous self-location and expanded body ownership.” We think that this characterization is still vague. Here is our proposal:

The current study of the Double Body Effect provides a partial but more precise phenomenological *simulation* of heautoscopy. Based on our experimental results, we suggest that (1) “bilocation” does not mean double self-location. Rather, once it is recognized that self-location and body-location are different phenomena, “bilocation” can be better specified as the experiences of double body ownership *and* double body-location. That is, it is *possible* that to experience “bilocation” is to experience the Double Body Effect. (2) While a subject can experience double body ownership and double body-location at the same time, this does *not* mean that there are two self-locations in the Double Body Effect. The CBT data of Experiments 2–4 suggest that the participants’ sense of self-location remained singular during the illusion. Thus, it can be hypothesized that the singular sense of self-location (as well as the singular sense of being a self) may stay intact in heautoscopy, unless proven otherwise. This can explain the singularity of “I” in many heautoscopic patients’ reports, including the one who claimed that “I am at both positions at the same time.”^62^^,^^63^ (3) In contrast to the sense of double body-location, participants in our experiments experienced only one 1PP-location during the Double Body Effect, either in between the two avatars (Experiment 2), or behind the two avatars (Experiment 3), or switching between them (Experiment 4). Hence, our data suggest that the participants’ sense of self-location was closely linked to their sense of 1PP-location. This supports the conjecture that heautoscopy might be the same in this regard. Finally, **(4)** the data of Experiment 4 can serve as a model for understanding the phenomenon of “perspective-switching” experienced by heautoscopic patients.

Although these points are preliminary, they are supported by the empirical data reported in this study. More studies are called for to see whether the Double Body Effect can be induced by different combinations of multisensory stimulations, such that these points may be confirmed in various ways. Also, more studies are required to verify how well these points capture the phenomenology of heautoscopy. Therefore, we are proposing them only as useful hypotheses for future research. Still, to the best of our knowledge, the Double Body Effect provides by far the closest and repeatable simulation of the phenomenology of heautoscopy. It is safe to say that we are finally beginning to make sense of this syndrome. Investigating the neural mechanisms of the Double Body Effect, we think, will help in the search for the neural mechanisms of heautoscopy.

### Conclusion

Drawing on the integration of proprioceptive-vestibular and visual information in virtual reality, our study provides a novel way of investigating BSC. For future research, we suggest that various combinations of proprioceptive-vestibular and visual stimulations can be developed to further investigate the Double Body Effect. We think that this experimental approach has great potential. It will contribute to a more precise comprehension of heautoscopy, and more generally, to a deeper understanding of BSC, including the phenomenological features of different types of BSC and their neural bases. Finally, what does the Double Body Effect tell us about the relation between body and self? Both body ownership and body location are basic types of BSC, and hence they illustrate and support the contemporary consensus that there is a close link between self and body. Our study is in accord with the view that the most basic sense of self, that is, the sense that each of us is a subject of conscious experience, is firmly rooted in bodily experiences. However, this does not mean that the self and body are the same thing. On the one hand, since it is empirically possible for healthy subjects to experience double body ownership and double body-location, these two types of BSC are, in fact, more flexible than most researchers have considered so far. On the other hand, we have suggested that self-location and body-location are not the same experiences. 1PP-location and body-location are different, too. Our claim is not that body-location is totally unrelated to self-location. Rather, our view is that body-location and 1PP-location are interrelated but distinct factors that jointly constitute the sense of self-location. Overall, our study shows that the relation between self and body is actually more complicated than both traditional dualists and contemporary reductionists have suggested. More interdisciplinary studies are required to clarify the complex self-body relationship.

### Limitations of the study

We would like to point out a few limitations of our experiments. First, the Bosu ball method caused the participants to constantly wobble, which helped induce the Double Body Effect. However, one drawback of this method was that, since the wobbling was mostly involuntary and hence not fully under the participants’ or the experimenters’ control, it was difficult to make sure that every participant received exactly the same numbers of proprioceptive-vestibular and visual stimuli across different conditions. Our experiments mainly relied on VR programming that provided a temporal-spatial match between visual feedback and participants’ actual movements in the synchronous conditions, and a discrepancy between them in the asynchronous conditions.

Second, we did not tease out the respective contributions of active and passive movements in this study. Hence, the roles they played in the Double Body Effect remain unclear. Further study is needed to dissect the significance of passive and active motor commands on this effect.

Third, in this study, the participants’ sense of body location and their sense of self-location were measured by different methods. While our questionnaires recorded participants’ responses regarding their body location, we conducted the CBT to measure their sense of self-location. A consequence of this approach was that the body-location results and the CBT data were in different formats. The structures of these two sets of data were heterogeneous, such that direct comparisons between them cannot be provided.

Fourth, Experiment 5 was designed to address the potential worry that the results of Experiments 2–4 only demonstrated a single-body effect that alternated between the two avatars. This experiment was similar to Experiment 2 in that the participants’ visual 1PP was set in the middle of the two avatars. Thus, strictly speaking, we only handled the issue with regard to Experiment 2, i.e., the 1PP conditions. We acknowledge that it would be better if more experiments like Experiment 5 were conducted to address similar worries with regard to the 3PP conditions (Experiment 3) and the 1PP-switching conditions (Experiment 4).

Finally, while we claim that our experimental results provide a useful and repeatable simulation of the phenomenology of heautoscopy, we are fully aware that this simulation is partial and preliminary. In future research, it should be taken into consideration that heautoscopic experience is often described as patients and their *doppelgänger* facing each other. Our designs in this study did not incorporate this aspect. Hence, to make progress, one possible design would be to immerse participants in a virtual environment in which two identical avatars face each other. Then, one can apply sensorimotor or proprioceptive-vestibular manipulations to see whether the Double Body Effect can be induced. Also, clinical neurology reports that “heautoscopy is often associated with the experience of sharing thoughts, words, or actions”^63^ (cf. also^60^). Thus, it would be significant to see whether the Double Body Effect can be induced by participants experiencing being embodied in two identical avatars such that the avatars not only face each other but also interact with each other.

## Resource availability

### Lead contact

Further information and requests for resources should be directed to and will be fulfilled by the lead contact, Sufen Chen (sufchen@mail.ntust.edu.tw).

### Materials availability

• This study did not generate any new unique reagents.
• The authors confirm that all data generated or analyzed during this study are included in this published article.
• Primary data and supplementary figures are provided in the supplemental information.

### Data and code availability

• The study has not been preregistered.
• This article does not report the original code.
• All data reported in this article will be shared by the lead contact upon request.
• Any additional information required to re-analyze the data reported in this article is available from the lead contact upon request.

## Acknowledgments

The authors would like to thank Ssu-Ching Huang for her assistance in our lab. We would also like to thank professor Chen-Gia Tsai from the Graduate Institute of Musicology for the SCR equipment. Finally, this study was supported by 10.13039/100020595National Science and Technology Council, Taiwan (10.13039/100020595NSTC 112-2410-H-002-103-MY2).

## Author contributions

C. L. designed all experiments; W.-K. L. provided VR technology; S. C. provided lab space and equipment; W.-H. L., W.-K. L., and B.-Y. C. built VR models; B.-Y. C., J.-R. L., and W.-H. L. conducted the experiments; S. C., J.-R. L., and Y.-T. L. analyzed the data; C. L. interpreted the data; C. L. and Y.-T. L. wrote the article.

## Declaration of interests

The authors declare no competing interests.

## STAR★Methods

### Key resources table

**Table:** 

REAGENT or RESOURCE	SOURCE	IDENTIFIER
**Deposited data**		

Raw and analyzed data	This paper and supplemental information	This paper and supplemental information

**Software and algorithms**		

Unity 3D	https://unity.com	https://unity.com
3ds Max	https://www.autodesk.com/products/3ds-max/overview	https://www.autodesk.com/products/3ds-max/overview
MANUS Core version 1.9.0	https://docs.manus-meta.com/2.5.0/Software/	https://docs.manus-meta.com/2.5.0/Software/
R version 4.5.1	https://www.r-project.org/	https://www.r-project.org/

### Experimental model and study participant details

#### Participants

We recruited a total of 209 participants who provided 196 valid data. Experiment 1 (19 females, mean age = 23.45 ± 4.06 years), Experiment 2 (21 females, mean age = 24.20 ± 5.37 years), Experiment 3 (22 females, mean age = 23.68 ± 4.53 years), and Experiment 4 (16 females, mean age = 24.20 ± 3.83 years) each involved a different group of 40 participants. Experiment 5 involved a different group of 36 participants (10 females, mean age = 26.91 ± 6.30 years). All participants were between 18–46 years old. Using G∗Power v. 3.1.9.7, we performed the power analysis to detect effects greater than *η*_*p*_^2^ = 0.06.^75^ To reach the power effect of 0.8, the standard value of psychological studies, each experiment should include more than 34 participants. This study was conducted in accordance with the Declaration of Helsinki, and the APA ethical standards were followed in the conduct of this study. This study was approved by the Research Ethics Committee of National Taiwan University (NTU-REC: 202212HM048).

#### Consent to participate

Informed consent to participate in the experiments was obtained from all individual participants included in the reported studies.

### Method details

#### Equipment and software

We used a head-mounted display (HMD, HTC VIVE PRO STARTER KIT/per eye 1440∗1600 image resolution, wide 110° field of view, and an ultra-smooth 90 Hz refresh rate) to display a virtual scene. Seven VR trackers (VIVE Tracker (2.0)/Indicator: IR LED) were used in the experiments to capture the participant’s full-body movements. Avatars were constructed using 3ds Max, Unity game engine software and VR SDK. To achieve realistic visual effects, we used 3ds Max for modeling, texturing, and meshing. We also used the rendering effects and dynamic lighting effects of Unity software when designing the virtual environment. The avatar and the environment were programmed according to 1:1 equal size ratio between the virtual world and the real world. The calibration between participants and avatars was carried out via MANUS Core 1.9.0. to achieve temporal and spatial matches. If any participants failed the calibration, their data were removed from our records.

#### Questionnaires

Our questionnaires were adapted from the standardized embodiment questionnaire developed by Peck and Gonzalez-Franco^76^ and Gonzalez-Franco and Peck.^77^ Questionnaires were administered in Experiments 1–4. We used a Likert scale from “strongly disagree” (−3) to “strongly agree” (+3). After each experiment, the participants were informed that they would orally answer a questionnaire presented on their HMD. They were advised to give answers based on their spontaneous subjective feeling rather than on thinking or reasoning. The questionnaires were in Chinese when presented to the participants. Tables 2 and 3 present the English translations. The order of questions was randomly distributed. The questionnaire of Experiment 1 (Table 2) consisted of 7 items, measuring the experiences of body ownership, proprioceptive-vestibular referral, agency, and body-location, plus a control question. The questionnaire of Experiments 2–4 (Table 3) consisted of 13 items, measuring the experiences of body ownership, proprioceptive-vestibular referral, agency, and body-location with respect to the right avatar, the left avatar and both avatars, plus a control question. If any participants answered positively to the control question in any of the trials, they were regarded as failing the control, and their data were removed from our records.

#### Self-location measurement: Color ball task (CBT)

We designed the CBT as a way of measuring the sense of self-location. Participants saw five 3D billiard balls with different numbers, appearing one at a time, spaced evenly apart in front of them. From left to right, the numbers were 5, 2, 7, 3, 6 (Figure S1). In Experiment 1, Ball-7 was set directly in the front of the participant’s visual 1PP. In Experiments 2–4, Ball-5 was located at the front-left of the left avatar. Ball-2 was directly in front of the left avatar. Ball-7 was in front of the participant’s visual 1PP, that is, between Ball-2 and Ball-3. Ball-3 was located directly in front of the right avatar. Finally, Ball-6 was at the front-right of the right avatar. Participants were told beforehand that five billiard balls would appear one at a time, and that each would appear twice. After that, they were instructed to answer the question: “Which ball do you feel to be closest to you?” Multiple choices were allowed. The avatars would disappear during the CBT. The participant did CBT twice in Experiments 1–4: before stepping onto the Bosu ball (pre-test) and after the experiments (post-test). We hypothesized that participants’ answers would be based on their sense of self-location before and after the experimental manipulations.

#### Skin conductance responses (SCR)

To record participants' skin conductance responses (SCR), we used a Data Acquisition Unit Biopac MP35 (Goleta, USA). We attached two single-use foam electrodes (Covidien, Inc., Mansfield, USA) on the participant’s nape.^78^ Each was placed 2 cm from the cervical vertebrae on each side, as other locations such as fingertips were not available during the Bosu ball exercise. SCR was measured by presenting threats: a virtual circular saw blade flying with noise toward the avatar in Experiment 1 (Figure 1D), or one of the two avatars in Experiment 5 (Figure 9C), or two virtual circular saw blades flying with noise toward both avatars in Experiments 2–4 (Figures 3C and 5C). The blades disappeared after they had touched the abdomen of the avatars for 4 s. To analyze SCR, the sampling rate was set at 200 samples per second, and the Hardware Filter was set to 20 KHz Lowpass. Data were analyzed with the Biopac software AcqKnowledge v. 3.7.7. We followed the standard procedure and identified the amplitude of SCR as the difference between the maximal and minimal values of the responses within 6 s of the threat. We excluded all the data of outliers if their SCR signals showed no amplitude or were lost during the threat procedures. Totally, 13 data were excluded from Experiment 5.

#### Experimental setup

Before the experiments began, participants were instructed to put on an orange jacket, and then an HMD that enabled them to look in any direction within the virtual environment. They wore seven VR motion trackers, including one on each hand, one on each upper arm near the elbow, one on each ankle, and one in the back waist position (Figure 1A). These motion trackers mapped the participants’ full-body movements from the real world onto the VR environment in real time with pinpoint accuracy. The avatar was programmed so that it could move synchronously with the participants’ real body movements. This recreated real-life movements in the virtual environment. Participants were immersed in a virtual gym where they saw and controlled one or two life-sized avatars. Their visual 1PP in the virtual gym was set to correspond to the position and orientation of the HMD that they wore in the real world. A Bosu ball (semicircular balance ball) was put in front of them. We attached a motion tracker to the Bosu ball such that it correlated perfectly with a virtual Bosu ball that the participants saw in front of the avatar. Two quad canes were set beside the Bosu ball in case the participants needed assistance to balance. The real quad canes were also correlated with two virtual quad canes that the participants saw in the virtual gym.

When the experiments started, participants were instructed to step onto the virtual Bosu ball in front of them. Since the virtual Bosu ball was correlated with a real Bosu ball in the lab, they would actually step onto the real Bosu ball at the same time. This would trigger their sense of proprioception and their sense of balance, and immediately caused them to wobble involuntarily to maintain balance. We programmed a virtual mirror in the gym, so the participants would see the avatar when they looked straight ahead. In the single body and 1PP conditions (Experiment 1), when the participants looked down at their body, instead of seeing their real body, they would see a life-sized avatar in an orange jacket, vivid and with shadows, standing on a Bosu ball in the virtual gym (Figures 1B and 1C). In the double body conditions (Experiments 2–5), when participants looked straight down, they would not see their body as usually expected. Rather, they would see two identical avatars standing on Bosu balls side by side. In Experiments 2 and 5, participants’ visual 1PP was set in the middle between the two avatars (Figures 3A, 9A, and 9B). In Experiment 3, the 1PP was set in the middle but about 60 cm behind the avatars (Figure 5A). This created a 3PP-experience, that is, the two avatars were seen from a short distance as if looking at other people’s bodies. In Experiment 4, participants’ 1PP was set to switch back and forth between the two avatars every 20 s (Figures 7A and 7B). See below for more details.

#### Procedure – Experiment 1: 1PP, single body conditions, Sync. vs. Async

Participants wore goggles and an orange jacket, then stood on a Bosu ball (Figure 1A). Via the HMD, participants saw a life-sized avatar in an orange jacket from the 1PP. After calibration, participants were told to move their head, hands and legs freely for 30 s, followed by the CBT pre-test. In the synchronous condition, participants were instructed to step onto the virtual Bosu ball in front of them and were required to stay on it throughout the experiment. When they looked down, they would see the avatar standing on a Bosu ball (Figure 1B). When they looked straight ahead, they would see the avatar in a mirror (Figure 1C). It took about 10 s for the participants to reach initial balance. As they continued to wobble involuntarily, they were instructed to (1) move their body freely for 80 s, then (2) look down for 40 s, (3) swing their arms for 80 s, and (4) move freely for another 80 s. During these procedures, participants watched the avatar doing exactly the same movements simultaneously. After that, the experimenters performed SCR measurement (Figure 1D), followed by the questionnaire and the CBT post-test. In the asynchronous condition, participants did not step onto the virtual Bosu ball, but stood on the ground the whole time. They saw the avatar from the 1PP but had no control of it at all, that is, they watched the avatar wobbling on its own on the virtual Bosu ball. Participants were instructed to (1) look down (while watching the avatar stepping onto the virtual Bosu ball on its own), (2) look straight ahead for 80 s (while watching the avatar wobbling on its own), (3) continue to look straight ahead for 40 s (while watching the avatar lowering its head and wobbling on its own), (4) continue to look straight ahead (while watching the avatar swinging its arms on its own), and take turns doing the following for 80 s (which were very different from the avatars’ movements seen via the HMD): cover their head with both hands (20 s), open their arms (20 s), bend their arms akimbo (20 s), and put their hands down (20 s). After these procedures, SCR was measured (Figure 1D), followed by the questionnaire and the CBT post-test.

#### Procedure – Experiment 2: 1PP, double body conditions, Sync. vs. Async

After calibration, participants were told to move their head, hands and legs freely for 30 s, followed by the CBT pre-test. In the synchronous condition, participants were instructed to step onto the virtual Bosu ball in front of them and were required to stay on it throughout the experiment. It took about 10 s for them to attain initial balance. Then they saw the avatar splitting into two identical avatars. The splitting process took 3 s. The participants’ visual 1PP was set in the middle between the two avatars. Therefore, when they looked down, they would see two identical avatars standing side by side on Bosu balls (Figure 3A). When they looked straight ahead, they would see the two avatars in the mirror (Figure 3B). As they continued to wobble, they were instructed to (1) move their body freely for 80 s, then (2) look down for 40 s, (3) swing their arms for 80 s, and (4) move freely for another 80 s. During these procedures, participants watched the two avatars doing exactly the same movements simultaneously. After that, the experimenters performed SCR measurement (Figure 3C), then administered the questionnaire and the CBT post-test. In the asynchronous condition, participants did not step onto the virtual Bosu ball, but stood on the ground the whole time. They saw the avatar from the 1PP but had no control of them at all. They were instructed to (1) look down (while watching the avatar stepping onto the virtual Bosu ball on its own), (2) look straight ahead (while watching the avatar splitting into two identical avatars with the participants’ 1PP located in the middle between the avatars, (3) look straight ahead for 80 s (while watching the two avatars wobbling on its own), (4) continue to look straight ahead for 40 s (while watching the avatar lowering its head and wobbling on its own), (5) continue to look straight ahead (while watching the avatar swinging its arms on its own) and take turns to do the following for 80 s (which were quite different from the avatars’ movements seen via the HMD): cover their head with both hands (20 s), open their arms (20 s), bend their arms akimbo (20 s), and put their hands down (20 s). After these procedures, SCR was measured (Figure 3C), followed by the questionnaire and the CBT post-test.

#### Procedure – Experiment 3: 3PP, double body conditions, Sync. vs. Async

The procedures of Experiment 3 were almost the same as those of Experiment 2. The only difference was that participants’ visual 1PP was set in the middle but about 60 cm behind the two avatars. Thus, they saw the two avatars from a short distance, that is, from the 3PP. If they looked straight down to where they usually saw their body, they would see nothing there. Rather, they would see two identical avatars about 60 cm in the front (Figure 5A). When they looked straight ahead, they would see the backs of the two avatars as well as the fronts of the two avatars in the mirror (Figure 5B).

#### Procedure – Experiment 4: 1PP-Switch, Double Body Conditions, Sync. vs. Async

The procedures of Experiment 4 were almost the same as those of Experiment 2. The only difference was that, when the avatar split into two identical avatars in both the synchronous and asynchronous conditions, participants’ 1 PP switched back and forth between the two avatars every 20 s throughout the experiment (Figures 7A–7D). The order of switching was evenly distributed: half of the participants started with their 1PP located at the left avatar and finished the experiment with their 1PP on the right avatar. The other half experienced the switching process in the opposite order.

#### Procedure – Experiment 5: 1PP, double body conditions, SCR only

The aim of Experiment 5 was to rule out the possibility that the results of Experiments 2–4 only demonstrated a single-body effect that alternated between the two avatars. The setting was very similar to the synchronous condition of Experiment 2, except that we performed only three rounds of SCR measurements. Condition A: both avatars were synchronous with respect to the participants, but only one avatar was threatened (randomly selected but evenly distributed) (Figure 9A). Condition B: one avatar was synchronous while the other squatted down and was asynchronous with respect to the participants (randomly selected and evenly distributed) (Figure 9B), and only the synchronous avatar was threatened. Condition C: the manipulation was the same as B, but only the asynchronous avatar was threatened. In all conditions, when one of the avatars was threatened, both would appear as standing (Figure 9C) to avoid possible confounding elements in the participants’ SCR responses. Every participant did all three rounds of SCR measurements, the order of which was random and evenly distributed. There was a 30-s break between every two measurements, and the participants remained on the Bosu ball throughout all of the procedures.

Based on previous literature of rubber hand illusions and full-body illusions, we expected that the results of Conditions A and B would be higher than the result of Condition C. Following Guterstam et al.,^26^ here is our key hypothesis: if there was no significant difference between Conditions A and B, it would support that the situations of A and B in fact reflect the same type of bodily experiences. That is, in Condition A, the double body condition, the participants experienced body ownership and body-location with respect to both avatars. This would then provide physiological evidence for the view that the effect induced in this study was not merely an alternating single-body phenomenon. Why? Since in Condition B only the synchronous avatar was threatened, one could reasonably expect that the likelihood for the threatened avatar to be experienced as the participant’s own body would be 100% or nearly as high. If the data collected in Experiments 2–4 revealed only an alternating single-body effect, one would expect that in Condition A the participants sometimes experienced ownership with respect to the avatar that was threatened but sometimes they did not. Then significantly lower values of SCR should be observed in Condition A as compared to Condition B, as the likelihood for the threatened avatar to be experienced as the participant’s own body in Condition A would be much lower than that in Condition B, say, only 50%. On the other hand, if there was no significant difference between Conditions A and B, it would support that the situations of A and B in fact reflect the same type of bodily experiences. Since there was no significant difference between the results of Conditions A and B (Figure 9D), the SCR data cannot be accommodated by the alternating single-body interpretation. Therefore, our experiments demonstrated a genuine Double Body Effect.

### Quantification and statistical analysis

#### Questionnaire – Experiments 1–4 and cross-experiment analyses

To test if the data fit the normal distribution, we conducted the Shapiro-Wilk test on the data of each experiment. Since the results showed that the data did not fit the normal distribution, we analyzed the data non-parametrically. Within the experiments, we compared each item between the synchronous and asynchronous manipulations using the Wilcoxon signed-rank test. Across the experiments, we first performed the Aligned Ranked Transform procedure, then conducted two sets of 3-way mixed-model ANOVAs. For the main effects and interaction effects identified by the ANOVAs, we conducted a Wilcoxon signed-rank test with a Bonferroni α correction for post-hoc analyses. Here are the results:

Experiment 1 (***N*** = 40): Q1: V = 541.5, *p* < 0.001, r = 0.702; Q2: V = 376.5, *p* = 0.011, r = 0.442; Q3: V = 349.5, *p* = 0.015, r = 0.425; Q4: V = 540, *p* < 0.001, r = 0.787; Q5: V = 580.5, *p* < 0.001, r = 0.810; Q6: V = 406.5, *p* < 0.001, r = 0.613; Q7: V = 101, *p* = 0.243, r = 0.140.

Experiment 2 (***N*** = 40): Q1: V = 513, *p* < 0.001, r = 0.603; Q2: V = 407.5, *p* = 0.002, r = 0.545; Q3: V = 579, *p* < 0.001, r = 0.807; Q4: V = 563, *p* < 0.001, r = 0.675; Q5: V = 421.5, *p* < 0.001, r = 0.621; Q6: V = 602, *p* < 0.001, r = 0.780; Q7: V = 541, *p* < 0.001, r = 0.689; Q8: V = 480.5, *p* < 0.001, r = 0.586; Q9: V = 610, *p* < 0.001, r = 0.786; Q10: V = 558, *p* < 0.001, r = 0.738; Q11: V = 435, *p* < 0.001, r = 0.693; Q12: V = 414.5, *p* = 0.001, r = 0.561; Q13: V = 50, *p* = 0.775, r = 0.046.

Experiment 3 (***N*** = 40): Q1: V = 648, *p* < 0.001, r = 0.728; Q2: V = 357, *p* < 0.001, r = 0.541; Q3: V = 662, *p* < 0.001, r = 0.841; Q4: V = 513, *p* < 0.001, r = 0.773; Q5: V = 414, *p* = 0.001, r = 0.521; Q6: V = 777, *p* < 0.001, r = 0.784; Q7: V = 598, *p* < 0.001, r = 0.761; Q8: V = 426, *p* = 0.002, r = 0.502; Q9: V = 615, *p* < 0.001, r = 0.806; Q10: V = 473, *p* < 0.001, r = 0.745; Q11: V = 520, *p* < 0.001, r = 0.703; Q12: V = 472, *p* < 0.001, r = 0.723; Q13: V = 47.5, *p* = 0.487, r = 0.061.

Experiment 4 (***N*** = 40): Q1: V = 415, *p* = 0.001, r = 0.522; Q2: V = 332, *p* = 0.100, r = 0.286; Q3: V = 640, *p* < 0.001, r = 0.783; Q4: V = 416, *p* = 0.001, r = 0.545; Q5: V = 420, *p* < 0.001, r = 0.709; Q6: V = 483, *p* < 0.001, r = 0.675; Q7: V = 398, *p* < 0.001,r = 0.663; Q8: V = 409, *p* < 0.001, r = 0.662; Q9: V = 442, *p* < 0.001, r = 0.708; Q10: V = 362, *p* = 0.008, r = 0.494; Q11: V = 392, *p* = 0.004, r = 0.476; Q12: V = 270, *p* = 0.052, r = 0.331; Q13: V = 294, *p* = 0.366, r = 0.167.

#### Cross-experiment analysis (first set)

First ANOVA: [Perspectival Difference] × [Synchronicity] × [Double Body Experience Items (DBEI)]. Main effects: Synchronicity (*F*_(1,546)_ = 285.326, *p* < 0.001, *η*_*p*_^2^(ART) = 0.343), and DBEI (*F*_(3,546)_ = 8.232, *p* < 0.001, *η*_*p*_^2^(ART) = 0.043). Interaction effects: Perspectival Difference × Synchronicity (*F*_(1,546)_ = 4.163, *p* = 0.042, *η*_*p*_^2^(ART) = 0.008), and Synchronicity × DBEI (*F*_(3,546)_ = 7.763, *p* < 0.001, *η*_*p*_^2^(ART) = 0.041).

Second ANOVA: [Perspectival Difference] × [Synchronicity] × [Right Body Experience Items (RBEI)]. Main effects: Perspectival Difference (*F*_(1,78)_ = 5.737, *p* = 0.019, *η*_*p*_^2^(ART) = 0.069), Synchronicity (*F*_(1,546)_ = 375.184, *p* < 0.001, *η*_*p*_^2^(ART) = 0.407), and RBEI (*F*_(3,546)_ = 8.421, *p* < 0.001, *η*_*p*_^2^(ART) = 0.044). Interaction effects: Synchronicity × RBEI (*F*_(3,546)_ = 7.334, *p* < 0.001, *η*_*p*_^2^(ART) = 0.039).

Third ANOVA: [Perspectival Difference] × [Synchronicity] × [Left Body Experience Items (LBEI)]. Main effects: Synchronicity (*F*_(1,546)_ = 351.586, *p* < 0.001, *η*_*p*_^2^(ART) = 0.392), and LBEI (*F*_(3,546)_ = 4.017, *p* = 0.008, *η*_*p*_^2^(ART) = 0.022). Interaction effects: Synchronicity × LBEI (*F*_(3,546)_ = 5.644, *p* = 0.001, *η*_*p*_^2^(ART) = 0.030).

#### Cross-experiment analysis (second set)

First ANOVA: [Perspectival Difference] × [Synchronicity] × [Body Ownership Item]. Main effects: Synchronicity (*F*_(1,585)_ = 378.374, *p* < 0.001, *η*_*p*_^2^(ART) = 0.393). Interaction effects: Perspectival Difference × Synchronicity (*F*_(2,585)_ = 7.099, *p* = 0.001, *η*_*p*_^2^(ART) = 0.024).

Second ANOVA: Perspectival Difference] × [Synchronicity] × [Referral Items]. Main effects: Synchronicity (*F*_(1,585)_ = 173.182, *p* < 0.001, *η*_*p*_^2^(ART) = 0.228). Interaction effects: none.

Third ANOVA: [Perspectival Difference] × [Synchronicity] × [Agency Items]. Main effects: Synchronicity (*F*_(1,585)_ = 662.898, *p* < 0.001, *η*_*p*_^2^(ART) = 0.537). Interaction effects: Perspectival Difference × Synchronicity (*F*_(2,585)_ = 5.184, *p* = 0.006, *η*_*p*_^2^(ART) = 0.017).

Fourth ANOVA: [Perspectival Difference] × [Synchronicity] × [Body-location Items]. Main effects: Perspectival Difference (*F*_(2,117)_ = 3.358, *p* = 0.038, *η*_*p*_^2^(ART) = 0.054), and Synchronicity (*F*_(1,585)_ = 212.848, *p* < 0.001, *η*_*p*_^2^(ART) = 0.267). Interaction effects: Perspectival Difference × Synchronicity (*F*_(2,585)_ = 6.886, *p* = 0.001, *η*_*p*_^2^(ART) = 0.023).

Fifth ANOVA: [Perspectival Difference] × [Synchronicity] × [both avatars vs. right avatar vs. left avatar]. Main effects: Synchronicity (*F*_(1,585)_ = 518.154, *p* < 0.001, *η*_*p*_^2^(ART) = 0.469). Interaction effects: Perspectival Difference × Synchronicity (*F*_(2,585)_ = 4.291, *p* = 0.014, *η*_*p*_^2^(ART) = 0.014).

#### CBT – Experiments 1–4

We summed the number of participants who chose each color ball and reported the descriptive statistics.

#### SCR – Experiments 1–4

We first conducted the Shapiro-Wilk test on the SCR data of each experiment and found that they did not fit the normal distribution. We then conducted the Wilcoxon signed-rank test to analyze the differences in SCR data between the synchronous and asynchronous conditions. Here are the results: Experiment 1 (***N*** = 40): V = 678, *p* < 0.001, r = 0.570. Experiment 2 (***N*** = 40): V = 640, *p* = 0.002, r = 0.489. Experiment 3 (***N*** = 40): V = 608, *p* = 0.007, r = 0.421. Experiment 4 (***N*** = 40): V = 557, *p* = 0.048, r = 0.312.

#### SCR – Experiment 5

The Shapiro-Wilk test on the SCR data showed that they did not fit the normal distribution. We therefore conducted the Friedman test to analyze the differences in SCR data between the A, B, and C conditions, and performed a Wilcoxon signed-rank test with a Bonferroni α correction for post-hoc analysis. Here are the results: Experiment 5 (***N*** = 36): Friedman test: χ^2^ = 9.389, *p* = 0.009, W = 0.130. Post-hoc analysis (α adjusted): Condition A vs. Condition B (V = 335, *p* = 1.000, r = 0.005); Condition B vs. Condition C (V = 497, *p* = 0.027, r = 0.429); Condition A vs. Condition C (V = 485.5, *p* = 0.051, r = 0.399).
