# Supplementary material for: Double body effect induced by integrating proprioceptive-vestibular and visual information
Source: iScience. 2025 Oct 21;28(11):113819. doi: 10.1016/j.isci.2025.113819 (PMC12636385; doi:10.1016/j.isci.2025.113819)
Supplement: Document S1. Figure S1 and Tables S1–S9 [file mmc1.pdf]

iScience, Volume *28*

Supplemental information

Double body effect induced by integrating proprioceptive-vestibular and visual information

Caleb Liang, Wen-Hsiang Lin, Wei-Kai Liou, Bo-Yu Chen, Jie-Rong Lin, Yen-Tung Lee, and Sufen Chen

Supplementary Information

PRIMARY DATA

Suppl. Table 1: Experiment 1 (*n* = 40), Questionnaire Descriptive Statistics

Sync.	Q1	Q2	Q3	Q4	Q5	Q6	Q7	SCR
**Max**	3.00	3.00	3.00	3.00	3.00	3.00	2.00	8.77
**3rd Q.**	2.00	2.00	2.00	2.00	2.00	2.00	-1.75	1.46
**median**	1.00	1.00	1.50	2.00	2.00	1.00	-2.00	0.64
**1st Q.**	0.00	-1.00	0.00	1.75	2.00	1.00	-3.00	0.41
**Min**	-2.00	-2.00	-3.00	-2.00	-2.00	-2.00	-3.00	0.06

Async.	Q1	Q2	Q3	Q4	Q5	Q6	Q7	SCR
**Max**	2.00	2.00	3.00	2.00	2.00	3.00	3.00	4.34
**3rd Q.**	1.00	1.25	1.25	1.00	1.00	2.00	-1.00	0.78
**median**	-1.00	0.50	1.00	-2.00	-1.00	1.00	-2.00	0.37
**1st Q.**	-2.00	-2.00	-1.00	-3.00	-2.00	-1.00	-3.00	0.18
**Min**	-3.00	-3.00	-3.00	-3.00	-3.00	-3.00	-3.00	0.04

Suppl. Table 2: Experiment 1 (*n* = 40), CBT Results

		Ball-5	Ball-2	Ball-7	Ball-3	Ball-6
**Sync.**	**Pre-test**	5%	2.5%	80%	17.5%	2.5%
	**Post-test**	0%	5%	82.5%	10%	5%
**Async.**	**Pre-test**	5%	5%	77.5%	22.5%	2.5%
	**Post-test**	0%	0%	87.5%	10%	2.5%

*Since multiple choices were allowed, the sum of percentages within one condition may exceed 100%.

Suppl. Table 3: Experiment 2 (*n* = 40), Questionnaire Descriptive Statistics

Sync.	Q1	Q2	Q3	Q4	Q5	Q6	Q7	Q8	Q9	Q10	Q11	Q12	Q13	SCR
**Max**	3.00	3.00	3.00	3.00	3.00	3.00	3.00	3.00	3.00	3.00	3.00	3.00	1.00	2.71
**3rd Q.**	2.00	2.00	2.25	2.00	2.00	3.00	2.00	2.00	3.00	2.00	2.00	2.00	-1.00	0.81
**median**	1.00	2.00	2.00	1.50	2.00	2.00	1.50	2.00	2.00	2.00	1.50	1.00	-3.00	0.54
**1st Q.**	-0.25	0.75	1.00	1.00	1.00	1.00	0.00	0.00	0.00	0.75	0.00	-0.25	-3.00	0.29
**Min**	-3.00	-3.00	-2.00	-3.00	-3.00	-3.00	-2.00	-2.00	-2.00	-3.00	-2.00	-3.00	-3.00	0.03

Async.	Q1	Q2	Q3	Q4	Q5	Q6	Q7	Q8	Q9	Q10	Q11	Q12	Q13	SCR
**Max**	3.00	3.00	3.00	3.00	3.00	3.00	3.00	3.00	3.00	3.00	3.00	3.00	2.00	2.30

**3rd Q.**	1.00	1.00	0.00	1.00	1.25	1.00	1.00	1.25	0.00	1.00	1.00	1.00	-1.00	0.47
**median**	-1.00	0.00	-1.50	-1.00	0.00	-1.00	-1.00	0.00	-1.00	-1.00	-1.00	-1.00	-3.00	0.28
**1st Q.**	-2.00	-2.00	-2.25	-2.00	-1.25	-2.25	-2.00	-2.00	-2.00	-2.00	-2.00	-2.00	-3.00	0.11
**Min**	-3.00	-3.00	-3.00	-3.00	-3.00	-3.00	-3.00	-3.00	-3.00	-3.00	-3.00	-3.00	-3.00	0.02

Suppl. Table 4: Experiment 2 (*n* = 40), CBT Results

		Ball-5	Ball-2	Ball-7	Ball-3	Ball-6
**Sync.**	**Pre-test**	0%	2.5%	87.5%	10%	0%
	**Post-test**	2.5%	2.5%	87.5%	10%	5%
**Async.**	**Pre-test**	5%	5%	77.5%	22.5%	2.5%
	**Post-test**	0%	0%	85%	22.5%	0%

*Since multiple choices were allowed, the sum of percentages within one condition may exceed 100%.

Suppl. Table 5: Experiment 3 (*n* = 40), Questionnaire Descriptive Statistics

Sync.	Q1	Q2	Q3	Q4	Q5	Q6	Q7	Q8	Q9	Q10	Q11	Q12	Q13	SCR
**Max**	3.00	3.00	3.00	3.00	3.00	3.00	3.00	3.00	3.00	3.00	3.00	3.00	1.00	2.45
**3rd Q.**	2.00	2.00	2.00	2.00	2.00	2.00	2.00	2.00	2.00	2.00	2.00	2.00	-2.00	0.65
**median**	1.00	1.00	2.00	1.00	1.00	1.00	1.00	1.00	2.00	1.00	1.00	1.00	-3.00	0.43
**1st Q.**	0.00	0.00	1.00	-1.00	0.00	1.00	-0.25	-1.00	1.00	-1.00	-1.00	-1.00	-3.00	0.27
**Min**	-3.00	-3.00	0.00	-3.00	-3.00	-2.00	-3.00	-3.00	-2.00	-3.00	-3.00	-3.00	-3.00	0.02

Async.	Q1	Q2	Q3	Q4	Q5	Q6	Q7	Q8	Q9	Q10	Q11	Q12	Q13	SCR
**Max**	3.00	3.00	2.00	2.00	3.00	2.00	3.00	3.00	3.00	1.00	3.00	3.00	1.00	1.54
**3rd Q.**	0.00	1.25	0.00	-1.00	1.00	-0.75	0.00	1.00	0.00	0.00	0.25	-1.00	-1.75	0.54
**median**	-2.00	0.00	-2.00	-2.00	0.00	-2.00	-2.00	0.00	-2.00	-1.00	-1.50	-2.00	-3.00	0.34
**1st Q.**	-2.25	-2.00	-2.25	-3.00	-2.00	-3.00	-3.00	-2.00	-3.00	-3.00	-3.00	-2.25	-3.00	0.16
**Min**	-3.00	-3.00	-3.00	-3.00	-3.00	-3.00	-3.00	-3.00	-3.00	-3.00	-3.00	-3.00	-3.00	0.02

Suppl. Table 6: Experiment 3 (*n* = 40), CBT Results

		Ball-5	Ball-2	Ball-7	Ball-3	Ball-6
**Sync.**	**Pre-test**	2.5%	12.5%	62.5%	17.5%	5%
	**Post-test**	0%	15%	62.5%	27.5%	5%
**Async.**	**Pre-test**	0%	7.5%	57.5%	40%	0%
	**Post-test**	0%	15%	67.5%	17.5%	0%

*Since multiple choices were allowed, the sum of percentages within one condition may exceed 100%.

Suppl. Table 7: Experiment 4 (*n* = 40), Questionnaire Descriptive Statistics

Sync.	Q1	Q2	Q3	Q4	Q5	Q6	Q7	Q8	Q9	Q10	Q11	Q12	Q13	SCR
**Max**	3.00	3.00	3.00	3.00	3.00	3.00	3.00	3.00	3.00	3.00	3.00	3.00	2.00	2.76
**3rd Q.**	2.00	2.00	2.25	2.00	2.00	2.00	2.00	2.00	2.00	2.00	2.00	2.00	-1.00	0.79
**median**	1.00	1.00	2.00	1.00	2.00	1.00	1.00	2.00	1.00	1.00	1.00	1.00	-2.00	0.35
**1st Q.**	-1.00	-2.00	1.00	-0.25	1.00	1.00	-1.00	0.75	0.00	-1.00	-0.25	-1.25	-3.00	0.22
**Min**	-3.00	-3.00	-3.00	-3.00	-3.00	-3.00	-3.00	-3.00	-3.00	-3.00	-3.00	-3.00	-3.00	0.03

Async.	Q1	Q2	Q3	Q4	Q5	Q6	Q7	Q8	Q9	Q10	Q11	Q12	Q13	SCR
**Max**	3.00	3.00	3.00	3.00	3.00	3.00	3.00	3.00	3.00	3.00	3.00	3.00	3.00	1.66
**3rd Q.**	1.00	2.00	1.00	1.00	1.00	1.00	1.00	1.00	1.00	1.00	1.00	1.00	-0.75	0.71
**median**	-1.00	-0.50	-2.00	0.00	0.00	-1.00	-0.50	0.00	-1.00	-1.00	0.00	0.00	-2.00	0.29
**1st Q.**	-2.25	-2.00	-3.00	-2.00	-2.00	-2.00	-2.00	-2.25	-3.00	-2.00	-2.00	-3.00	-3.00	0.09
**Min**	-3.00	-3.00	-3.00	-3.00	-3.00	-3.00	-3.00	-3.00	-3.00	-3.00	-3.00	-3.00	-3.00	0.02

Suppl. Table 8: Experiment 4 (*n* = 40), CBT Results

		Ball-5	Ball-2	Ball-7	Ball-3	Ball-6
**Sync.**	**Pre-test**	0%	5%	77.5%	17.5%	7.5%
	**Post-test**	0%	12.5%	72.5%	22.5%	5%
**Async.**	**Pre-test**	0%	2.5%	80%	15%	5%
	**Post-test**	0%	10%	75%	25%	0%

*Since multiple choices were allowed, the sum of percentages within one condition may exceed 100%.

Suppl. Table 9: Experiment 5 (*n* = 36), SCR Descriptive Statistics

Sync.	Condition A	Condition B	Condition C
**Max**	1.92	3.47	1.69
**3rd Q.**	0.66	0.66	0.40
**median**	0.36	0.40	0.21
**1st Q.**	0.15	0.15	0.10
**Min**	0.02	0.02	0.02

SUPPLEMENTARY FIGURES

Suppl. Figure 1. Self-location measurement: Color Ball Task (CBT)

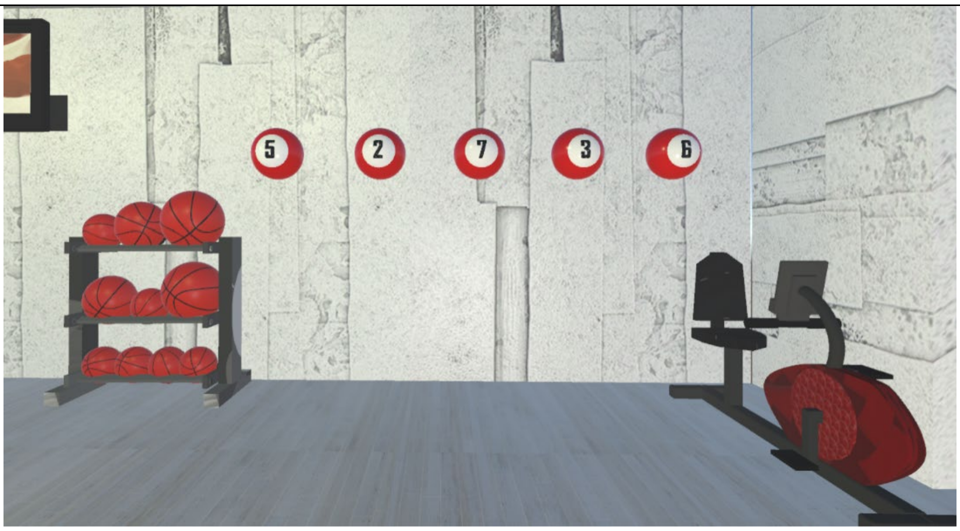

Suppl. Figure 1. Set-up of the Color Ball Test (CBT). In Experiments 1 ~ 4, the participants saw five 3D billiard balls with different numbers, appearing one at a time, spaced evenly apart in front of them. From left to right, the numbers were 5, 2, 7, 3, and 6.
